# *RAB11FIP5*-Deficient Mice Exhibit Cytokine-Related Transcriptomic Signatures

**DOI:** 10.4049/immunohorizons.2000088

**Published:** 2020-11-10

**Authors:** Dapeng Li, Todd Bradley, Derek W. Cain, Isabela Pedroza-Pacheco, Maria Aggelakopoulou, Robert Parks, Maggie Barr, Shi-Mao Xia, Richard Scearce, Cindy Bowman, Grace Stevens, Amanda Newman, Bhavna Hora, Yue Chen, Kristina Riebe, Yunfei Wang, Gregory Sempowski, Kevin O. Saunders, Persephone Borrow, Barton F. Haynes

**Affiliations:** * Duke Human Vaccine Institute, Duke University School of Medicine, Durham, NC 27710; † Department of Medicine, Duke University School of Medicine, Durham, NC 27710; ‡ Nuffield Department of Clinical Medicine, University of Oxford, OX3 7FZ Oxford, United Kingdom; § Department of Immunology, Duke University School of Medicine, Durham, NC 27710; ¶ Department of Surgery, Duke University, Durham, NC 27710

## Abstract

Rab11 recycling endosomes are involved in immunological synaptic functions, but the roles of Rab11 family–interacting protein 5 (Rab11Fip5), one of the Rab11 effectors, in the immune system remain obscure. Our previous study demonstrated that *RAB11FIP5* transcripts are significantly elevated in PBMCs from HIV-1–infected individuals, making broadly HIV-1–neutralizing Abs compared with those without broadly neutralizing Abs; however, the role of Rab11FiP5 in immune functions remains unclear. In this study, a *RAB11FIP5* gene knockout (*RAB11FIP5*^−/−^) mouse model was employed to study the role of Rab11Fip5 in immune responses. *RAB11FIP5*^−/−^ mice exhibited no perturbation in lymphoid tissue cell subsets, and Rab11Fip5 was not required for serum Ab induction following HIV-1 envelope immunization, Ab transcytosis to mucosal sites, or survival after influenza challenge. However, differences were observed in multiple transcripts, including cytokine genes, in lymphocyte subsets from envelope-immunized *RAB11FIP5*^−/−^ versus control mice. These included alterations in several genes in NK cells that mirrored observations in NKs from HIV-infected humans expressing less *RAB11FIP5*, although Rab11Fip5 was dispensable for NK cell cytolytic activity. Notably, immunized *RAB11FIP5*^−/−^ mice had lower *IL4* expression in CD4^+^ T follicular helper cells and showed lower *TNF* expression in CD8^+^ T cells. Likewise, TNF-α production by human CD8^+^ T cells correlated with PBMC *RAB11FIP5* expression. These observations in *RAB11FIP5*^−/−^ mice suggest a role for Rab11Fip5 in regulating cytokine responses.

## INTRODUCTION

Rab11 GTPase is a key regulator of recycling endosome trafficking, phagocytosis, exocytosis, and cytokinesis (1, 2). A family of effector proteins involved in these biological pathways through interactions with Rab11 has been termed Rab11 family–interacting proteins (Rab11Fips) (3). These Rab11FIP molecules are grouped into two classes based upon their Rab11 binding domain structure: class I (Rab11Fip1/2/5) and class II (Rab11Fip3/4) (4). One of the effectors, Rab11Fip5, has been suggested to play critical roles in a variety of biological functions, including transcytosis of the polymeric IgR (5), as well as translocation of insulin in pancreatic β cells (6), GLUT4 in adipocytes (7), and V-ATPase in duct cells (8). In addition, in the nervous system, Rab11Fip5 is required for hippocampal long-term depression (9) and may be associated with autism (10). However, the roles of Rab11Fip5 and Rab11Fip5-associated recycling endosomes in the immune system have not been well characterized.

We recently found that the *RAB11FIP5* transcript is significantly elevated in PBMCs from HIV-1–infected individuals who make broadly neutralizing Abs (bnAbs) compared with those who do not make bnAbs (11). People developing bnAbs have higher *RAB11FIP5* transcripts in multiple cell subsets, including NK cells, plasmacytoid dendritic cells, CD8^+^ T cells, and CD4^+^ T cells. We have demonstrated that the elevated *RAB11FIP5* expression in NK cells was associated with profound dysfunction of NK cells (11), which play a critical role in controlling T follicular helper (Tfh) cell availability to provide help for humoral immune responses (11-13). However, the elevation in *RAB11FIP5* expression in other immune cell subsets may also have been indicative of alterations in their functional capacity that formed part of the unique immunological environment facilitating bnAb generation. Interestingly, we further demonstrated that overexpression of Rab11Fip5 in an NK cell line increased NK cell functions, including degranulation and cytokine production, indicating that the association of higher *RAB11FIP5* expression with NK cell dysfunction in HIV-infected individuals was not causative (11). Another study by Reefman and colleagues suggested that the cytokines IFN-γ and TNF-α are trafficked and secreted via Rab11-associated recycling endosome pathway in NK cells (14). These observations point to an important, but as yet poorly characterized, role for Rab11Fip5 in the function of NK cells and putatively also other immune system cell subsets.

In this study, we employed a constitutive *RAB11FIP5* gene knockout (KO) (*RAB11FIP5*^−/−^) mouse model, in which Rab11Fip5 expression was abolished by the deletion of exon 2 of the *RAB11FIP5* gene (9) to examine the role of Rab11Fip5 in vaccine-induced immune responses. Although Rab11Fip5 was not required for systemic or mucosal Ab responses to vaccination, NK cytotoxicity, or survival after influenza infection, mice with Rab11Fip5 deficiency exhibited a series of cytokine-related transcriptomic signatures in lymphocyte subsets after HIV-1 vaccination. Notably, we found that Rab11Fip5 is associated with TNF-α production in CD8^+^ T cells and IL-4 production in CD4^+^ T cells upon stimulation in both mice and humans, suggesting that Rab11Fip5 may be important for the regulation of cytokine responses.

## MATERIALS AND METHODS

### Mice

*RAB11FIP5*^fl/fl^ mice (C57BL/6-Rab11fip5^tm1Sud^/J; Jax no. 021776; The Jackson Laboratory, Bar Harbor, ME) and CMV-Cre mice [B6.C-Tg(CMV-cre)1Cgn/J; Jax no. 006054] were both on a C57BL/6 background. *RAB11FIP5*^−/−^ mice were generated by breeding the *RAB11FIP5*^fl/fl^ mice with CMV-Cre mice as described previously (9). Briefly, in the *RAB11FIP5*^fl/fl^ conditional mutant mouse, exon 2 of the *RAB11FIP5* gene was flanked by loxP sites. Homozygous *RAB11FIP5*^fl/fl^ were observed to be viable, fertile, and produce normal levels of Rab11Fip5 protein. When crossed with a Cre-expressing strain, the floxed exon 2 was excised, and therefore, the expression of functional *RAB11FIP5* mRNA was abolished. As shown in Supplemental Fig. 1, after F5, all the mice became a constitutive *RAB11FIP5*^−/−^ strain. All experiments were conducted with mice after F5, and they were performed following protocols approved by the Duke University Institutional Animal Care and Use Committee.

*RAB11FIP5*^fl/fl^ mice were genotyped using primers *RAB11FIP5*-16761 (5′-ACT GAA GGG GGT GAG CAA G-3′) and Rab11FIP5-16762 (5′-CCC TAC CTG GAA AGG AAA TGT-3′). CMV-Cre mice were genotyped using primers Cre-oIMR1084 (5′-GCG GTC TGG CAG TAA AAA CTA TC-3′), Cre-oIMR1085 (5′-GTG AAA CAG CAT TGC TGT CAC TT-3′), Cre-oIMR7338 (5′-CTA GGC CAC AGA ATT GAA AGA TCT-3′), and Cre-oIMR7339 (5′-GTA GGT GGA AAT TCT AGC ATC ATC C-3′). *RAB11FIP5*^−/−^ mice were genotyped using three sets of primers: the *RAB11FIP5* primer set, CMV-Cre primer set, and primers for detecting the deletion of exon 2 in the *RAB11FIP5* gene in the genome; these primers were TB11885 (5′-TGCTTGCCATGATCTGTCCT-3′) and TB11887 (5′-GAGGTTCCTCATTGTACACATGG-3′) (9). At the conclusion of the experiments, tissues were collected for genotype confirmation by quantitative PCR (qPCR) using a TaqMan probe for *RAB11FIP5* Mm00624247_m1 (the probe spans exon 2) and a TaqMan probe for the control gene GAPDH Mm99999915_g1. By the F5 generation of breeding, as shown in Supplemental Fig. 1, samples from the *RAB11FIP5*^−/−^ mouse were expected to have no bands for *RAB11FIP5* or CMV-Cre by PCR, a 442-bp band with the TB11885/TB11887 primers, and no expression detected by the *RAB11FIP5* TaqMan probe.

### Immunizations

The study protocols and all veterinarian procedures were approved by the Duke University Institutional Animal Care and Use Committee and were performed based upon standard operating procedures. Mice (6–10-wk-old, both male and female) were immunized three times at 2-wk intervals with CH505 transmitted founder (TF) chimeric SOSIP trimers (25 μg/mouse) formulated in a TLR 4 agonist adjuvant GLA-SE (5 μg/mouse). Blood samples were collected 1 wk before priming and 1 wk after each injection. Bone marrow, spleen, lymph nodes, and Peyer’s patch cells were harvested 1 wk after the final immunization. Phenotypic staining was performed using freshly harvested cells, and the remainder of the cells were frozen in liquid nitrogen for RNA sequencing (RNA-Seq), qPCR, and sorting.

### Flow cytometry sorting of mouse immune cell subsets

Cryopreserved mouse splenocytes were thawed and stained using the following Abs: FITC anti-CD4 (clone no. RM4-5, catalog no. 553047; 1:400 dilution; Becton Dickinson [BD]), PE anti-CD25 (clone no. 7D4, catalog no. 558642, 1:1000 dilution; BD), PE-CF594 anti-CD279 (PD-1) (clone no. J43, catalog no. 562523, 1:500 dilution; BD), biotin anti-CXCR5 (clone no. 2G8, catalog no. 551960, 1:100 dilution; BD), Alexa Fluor (AF) 700 anti-CD8 (clone no. 53-6.7, catalog no. 564983, 1:200 dilution; BD), Brilliant Violet (BV) 421 anti-CD127 (clone no. SB/199, catalog no. 566300, 1:100 dilution; BD), BV510 anti-CD3 (clone no. 145-2C11, catalog no. 563024, 1:100 dilution; BD), BV650 anti-NK1.1 (clone no. PK136, catalog no. 108740, 1:100 dilution; BioLegend), BV786 anti-B220 (clone no. RA3-6B2, catalog no. 563894, 1:200 dilution; BD), and streptavidin-AF647 (catalog no. 405237, 1:1500 dilution; BioLegend). B cells (CD3^−^B220^+^), NK cells (CD3^−^NKU^+^), CD8^+^ T cells (CD3^+^CD8^+^), Tfh cells (CD3^+^CD4^+^CD8^−^CD127^high^CD25^low^PD-1^+^CXCR5^+^), and T follicular regulatory (Tfr) cells (CD3^+^CD4^+^CD8^−^CD127^low^CD25^high^PD-1^+^CXCR5^+^) were sorted on a BD FACSAria Flow Cytometer.

### RNA-Seq

For RNA-Seq, immune cell populations were sorted into RLT Plus lysis buffer, and total RNA was purified using the RNeasy Micro Kit (QIAGEN). RNA samples were subjected to reverse transcription and amplification using the SMART-Seq Ultra Low v4 Kit (Clontech Laboratories). Two hundred picograms of amplified cDNA was prepared for Illumina sequencing using the Nextera XT Library Prep Kit (Illumina). Libraries were quantified using qPCR (Kapa Biosystems) and sequenced to a minimum depth of 25 million reads per sample (2 × 75 bp reads) on the Illumina NextSeq. After sequencing, fastq files were quality filtered and trimmed using Trim Galore and aligned to the human or mouse genome using Spliced Transcripts Alignment to a Reference (STAR). After reference alignment, read counts on each gene were quantified by HTSeq, and significant differentially expressed transcripts were determined by DeSeq2 R package.

### qPCR

To quantitate expression of Rab11Fip, cytokine, and chemokine receptor genes, RNA was reverse transcribed using the High-Capacity RNA-to-cDNA kit (Thermo Fisher Scientific), and qPCR was carried out in duplicate wells using the TaqMan probes for *RAB11FIP5* Mm00624247_m1, *CCR1L1* Mm00432606_s1, *IFNG* Mm99999071_m1, *IL4* Mm00445259_m1, *TNF* Mm00443258_m1, *RAB11FIP1* Mm01214977_m1, *RAB11FIP2* Mm01261268_m1, *RAB11FIP3* Mm00462418_m1, *RAB11FIP4* Mm00558604_m1, and *GAPDH* Mm99999915_g1. Expression of GAPDH was subtracted from *RAB11FIP5* to normalize for input, and the resulting △ cycle threshold values were log transformed to calculate relative expression.

### ELISA

In total, 2 μg/ml of protein in sodium bicarbonate buffer was incubated in sealed Nunc-absorp (Thermo Fisher Scientific) plates overnight at 4°C. The plates were then washed and blocked with SuperBlock for 1 h. Serially diluted mouse serum or vaginal lavage samples were added to the plate and incubated at room temperature for 90 min. Binding Abs were detected with 1:30,000 dilution of HRP-labeled anti-IgG Abs (catalog no. 1013-05; SouthernBiotech) or HRP-labeled anti-IgA Abs (catalog no. 1040-05; SouthernBiotech). HRP was detected with 3,3′,5,5′-tetramethylbenzidine. Binding titers were analyzed as area under curve (AUC) of the log-transformed concentrations.

### HIV-1 neutralization assays

Ab neutralization of HIV-1 was measured in TZM-bl cell-based assays as described previously (15-18). Results were read out as a reduction in luminescence units compared with control wells and reported as IC_50_ in micrograms per milliliter.

### Cell surface staining

For phenotypic analysis of mouse B cells, single suspensions of cells from spleen, lymph node, and bone marrow were stained using the following Abs: FITC anti-IgG1 (clone no. A85-1, catalog no. 553443, 1:1000 dilution; BD), FITC anti-IgG2 (clone no. R2-40, catalog no. 553399, 1:200 dilution; BD), FITC anti-IgG3 (clone no. R40-82, catalog no. 553403, 1:200 dilution; BD), PerCP-Cy5.5 anti-CD21 (clone no. 7E9, catalog no. 123416, 1:1000 dilution; BioLegend), PE anti-GL7 (clone no. GL7, catalog no. 561530, 1:2000 dilution; BD), PE-CF594 anti-CD93 (clone no. AA4.1, catalog no. 563805, 1:400 dilution; BD), PE-Cy5 anti-CD38 (clone no. 90, catalog no. 15-0381-82, 1:2000 dilution; eBioscience), PE-Cy7 anti-IgM (clone no. R6-60.2, catalog no. 552867, 1:400 dilution; BD), AF700 anti-CD19 (clone no. 1D3, catalog no. 565473, 1:400 dilution; BD), BV510 anti-IgD (clone no. 11-26C.2a, catalog no. 563110, 1:400 dilution; BD), BV605 anti-CD95 (clone no. Jo2, catalog no. 740367, 1:400 dilution; BD), BV650 anti-B220 (clone no. RA3-6B2, catalog no. 563893, 1:400 dilution; BD), BV711 anti-CD138 (clone no. 281-2, catalog no. 563193, 1:1000 dilution; BD), and BV786 anti-CD23 (clone no. B3B4, catalog no. 563988, 1:1000 dilution; BD). For Ag-specific B cell phenotyping, single suspensions of cells from spleen, lymph node, bone marrow, and Peyer’s patch were stained using the B cell panel described above, and an Ag-specific B cell subset was detected by using the combination of tetramerized, AF647-, and BV421-conjugated HIV-1 envelope (Env) CH505 TF-derived gp120 proteins. LIVE/DEAD Near IR was used to discriminate live from dead cells.

For phenotypic analyses of mouse T and NK cells, single suspensions of cells from spleen, lymph node, and bone marrow were stained using the following Abs: FITC anti-CD4 (clone no. RM4-5, catalog no. 553047, 1:800 dilution; BD), PerCp/Cy5.5 anti-CD49b (clone no. DX5, catalog no. 108916, 1:200 dilution; BioLegend), PE anti-CD25 (clone no. 7D4, catalog no. 558642, 1: 2000 dilution; BD), PE-CF594 anti-CD279 (PD-1) (clone no. J43, catalog no. 562523, 1:1000 dilution; BD), PE-Cy5 anti-TER119 (clone no. TER119, catalog no. 116210, 1:200 dilution; BioLegend), PE-Cy7 anti-CD62L (clone no. MEL-14, catalog no. 560516, 1:1000 dilution; BD), biotin anti-CXCR5 (clone no. 2G8, catalog no. 551960, 1:200 dilution; BD), AF700 anti-CD8 (clone no. 53-6.7, catalog no. 564983, 1:400 dilution; BD), BV421 anti-CD127 (clone no. SB/199, catalog no. 566300, 1:200 dilution; BD), BV510 anti-CD3 (clone no. 145-2C11, catalog no. 563024, 1:200 dilution; BD), BV605 anti-Th1.2 (clone no. 53-2.1, catalog no. 563008, 1:1000 dilution; BD), BV650 anti-NK1.1 (clone no. PK136, catalog no. 108740, 1:200 dilution; BioLegend), BV711 anti-CD44 (clone no. IM7, catalog no. 563971, 1:1000 dilution; BD), BV786 anti-B220 (clone no. RA3-6B2, catalog no. 563894, 1:400 dilution; BD), and streptavidin-AF647 (catalog no. 405237, 1:3000 dilution; BioLegend). LIVE/DEAD Near IR was used to discriminate live from dead cells. Data were acquired on a BD LSR II flow cytometer and analyzed using FlowJo version 10.

### Intracellular staining of murine NK and T cells

For mouse NK cell intracellular staining and degranulation analysis, spleen cells isolated from mice were stimulated with 500 ng/ml PMA and 5 μg/ml ionomycin, 10 ng/ml IL-12 + 50 ng/ml IL-15 + 50 ng/ml IL-18, or YAC-1 target cells in the presence of anti-CD107a Ab and GolgiStop protein transport inhibitor (catalog no. 554724; BD) for 2h. Cells were then stained with Aqua Viability Dye (catalog no. L34957; Thermo Fisher Scientific) and with surface marker Abs FITC anti-NKG2D (clone no. C7, catalog no. 115711, 1:50 dilution; BioLegend), allophycocyanin–Fire 750 anti-NKp46 (clone no. 29A1.4, catalog no. 137632, 1:50 dilution; BioLegend), and BV650 anti-NK1.1 (clone no. PK136, catalog no. 108740, 1:100 dilution; BioLegend). The cells were then fixed with 2% paraformaldehyde and stained with PE–Dazzle 594 anti–granzyme B (clone no. QA16A02, catalog no. 372216, 1:200 dilution; BioLegend), allophycocyanin anti-perforin (clone no. S16009A, catalog no. 154304, 1:200 dilution; BioLegend), and BV711 anti–IFN-γ (clone no. XMG1.2, catalog no. 505836, 1:100 dilution; BioLegend) Abs in permeabilization buffer (catalog no. 88-8824; Invitrogen).

For mouse T cell intracellular staining, spleen cells isolated from either *RAB11FIP5*^−/−^ mice or *RAB11FIP5*^fl/fl^ control mice were stimulated with 500 ng/ml PMA and 5 μg/ml ionomycin in the presence of GolgiStop Protein Transport Inhibitor (catalog no. 554724; BD) for 4 h. Cells were stained using the following Abs: PE-Cy7 anti-CD25 (clone no. PC61, catalog no. 102016, 1:500 dilution; BioLegend), allophycocyanin anti-CXCR5 (clone no. 2G8, catalog no. 560615, 1:100 dilution; BD), AF700 anti-CD4 (clone no. OKT4, catalog no. 317426, 1:500 dilution; BioLegend), BV421 anti-CD127 (clone no. SB/199, catalog no. 566300, 1:100 dilution; BD), BV510 anti-CD3 (clone no. 145-2C11, catalog no. 563024, 1:100 dilution; BD), and BV605 anti-CD279 (PD-1) (clone no. J43, catalog no. 563059, 1:500 dilution; BD). The cells were fixed with 2% paraformaldehyde and stained with AF488 anti–IL-4 (clone no. 11B11, catalog no. 557728, 1:100 dilution; BD), biotin anti–IL-5 (clone no. TRFK4, catalog no. 504402, 1:100 dilution; BD), and streptavidin-AF594 (catalog no. 405240, 1:3000 dilution; BioLegend) Abs in permeabilization buffer (catalog no. 88-8824; Invitrogen). LIVE/DEAD Near IR was used to discriminate live from dead cells. Data were acquired on a BD LSR II flow cytometer and analyzed using FlowJo version 10.

For the CD4 T cell–spleen cell coculture experiments, CD4^+^ T cells were isolated from spleen cells of either *RAB11FIP5*^−/−^ mice or *RAB11FIP5*^fl/fl^ control mice using EasySep Mouse CD4 T Cell Isolation Kit (catalog no. 19852; STEMCELL Technologies). Isolated *RAB11FIP5*^−/−^ or *RAB11FIP5*^fl/fl^ control CD4 T cells were labeled with CellTrace Yellow dye (catalog no. C34573; Thermo Fisher Scientific) and mixed in a 1:1 ratio with *RAB11FIP5*^fl/fl^ control mice spleen cells as shown in Fig. 5E, 5F. The *RAB11FIP5*^−/−^ CD4 T cell–control spleen cell mixture, as well as the control CD4 T cell–control spleen cell mixture, were cocultured overnight, stimulated with Cell Activation Cocktail (catalog no. 423304, 1:500 dilution; BioLegend) either for 4 h or overnight, and incubated with the same Abs indicated above for measuring IL-4 production.

### Intracellular staining of human T cells

For intracellular TNF-α staining of primary human T cells, PBMCs from 26 HIV-infected subjects were stimulated with 125 ng/ml anti-human CD3 (catalog no. MAB100; R&D Systems) and 250 ng/ml anti-human CD28 (catalog no. 16-0289-85; Thermo Fisher Scientific) in 96-well round-bottom plates (catalog no. 3799; Corning) for 72 h. In the last 5 h of culture, 85 ng/ml PMA (catalog no. P8139; Sigma), 250 ng/ml ionomycin (catalog no. I0634; Sigma), and 3 μg/ml brefeldin (catalog no. 00-4506; Thermo Fisher Scientific) were added. Cells were then stained with Aqua Viability Dye (catalog no. L34957; Thermo Fisher Scientific) and with surface marker Abs PE–Texas Red anti-CD3 (clone no. 7D6, catalog no. MHCD0317, 1:100 dilution; Thermo Fisher Scientific), BV650 anti-CD4 (clone no. OKT4, catalog no. 317436, 1:100 dilution; BioLegend), BV510 anti-CD14 (clone no. M5E2, catalog no. 301842, 1:100 dilution; BioLegend), PE-Cy7 anti-CD19 (clone no. SJ25C1, catalog no. 363012, 1:100 dilution; BioLegend), allophycocyanin anti-CD21 (clone no. Bu32, catalog no. 354906, 1:100 dilution; BioLegend), allophycocyanin–Cy7 anti-CD24 (clone no. ML5, catalog no. 311132, 1:50 dilution; BioLegend), BV605 anti-CD27 (clone no. O323, catalog no. 302830, 1:100 dilution; BioLegend), BV785 anti-CD38 (clone no. HIT2, catalog no. 303530, 1:50 dilution; BioLegend), and PE anti-CD43 (clone no. 1G10, catalog no. 560199, 1:500 dilution; BD). The cells were then fixed and permeabilized with BD Fixation/Permeabilization Solution Kit (catalog no. 554714; BD) and stained with AF700 anti–Granzyme B (clone no. GB11, catalog no. 560213, 1:200 dilution; BD), BV421 anti–IL-10 (clone no. JES3-9D7, catalog no. 501422, 1:100 dilution; BioLegend), FITC anti–IL-6 (clone no. MQ2-13A5, catalog no. 501104, 1:50 dilution; BioLegend), PerCP–eFluor 710 anti–Ki-67 (clone no. SolA15, catalog no. 46-5698-82, 1:70 dilution; Thermo Fisher Scientific), and BV711 anti–TNF-α (clone no. Mab11, catalog no. 502940, 1:50 dilution; BioLegend). Data were collected on a BD LSR Fortessa X-20 and analyzed using FlowJo version 10.

### Chromium-51 release assay for NK cell killing

NK cell cytotoxicity was measured with a standard chromium-51 (^51^Cr) release assay, using enriched mouse NK cells as effector cells and ^51^Cr-labeled YAC-1 as target cells. Target cells were labeled with Na_2_^51^CrO_4_ (PerkinElmer) at 250 μCi/ml for 2 h at 37°C. After washing three times, cells were mixed with effector cells at final E:T ratios of 10:1, 3:1, and 1:1 in triplicate wells in flexible 96-well round-bottom plates (catalog no. 1450-401; PerkinElmer). The plates were inserted into flexible 96-well plate cassettes (catalog no. 1450-101; PerkinElmer), sealed, and incubated at 37°C for 4 h. After incubation, cells were pelleted by centrifugation, and from the top of the well, 25 μl of supernatant was carefully added to rigid 96-well isoplates (catalog no. 1450-514; PerkinElmer) containing 150 μl of Ultima Gold LSC Cocktail (catalog no. L8286; Sigma). The plates were inserted into rigid 96-well plate cassettes (catalog no. 1450-105; PerkinElmer), sealed, and counted in a PerkinElmer MicroBeta TriLux 1450 counter. ^51^Cr-labeled target cells without effector cells were analyzed as a spontaneous release control, and ^51^Cr-labeled target cells mixed with detergent (2% Triton X-100) were used as a maximum release control. The percentages of specific lysis were calculated as follows: Percentage of Specific Lysis (^51^Cr Release Percentage) = [(Experimental Release – Spontaneous Release)/(Maximum Release – Spontaneous Release)] × 100.

### Influenza virus infection

*RAB11FIP5*^−/−^ mice (*n* = 20) and *RAB11FIP5*^fl/fl^ control mice (*n* = 20) were intranasally challenged with a high- (10^6^ median tissue culture infective dose [TCID50]) or low-dose (10^4^ TCID50) influenza H3N2 X-31 virus. Survival and body weight were monitored daily for 10 d. Animals that reached the body weight cutoff (80% of original weight) were euthanized.

### Statistical analyses

Prism 8 (GraphPad Software) and R were used to plot the data. For statistical analysis in mouse immunization studies, the Wilcoxon rank sum exact test (Mann–Whitney *U* test) was performed using SAS 9.4 (SAS Institute, Cary, NC) or Prism 8. AUC were calculated by Prism 8. Pearson correlation analysis was used to test the correlation between *RAB11FIP5* mRNA in PBMC and TNF-α expression in human CD3^+^CD4^−^ T cells. Thep values <0.05 were considered significant.

## RESULTS

### Transcriptome analysis of immune cell subsets in immunized RAB11FIP5^−/−^ mice

**FIGURE 1.**
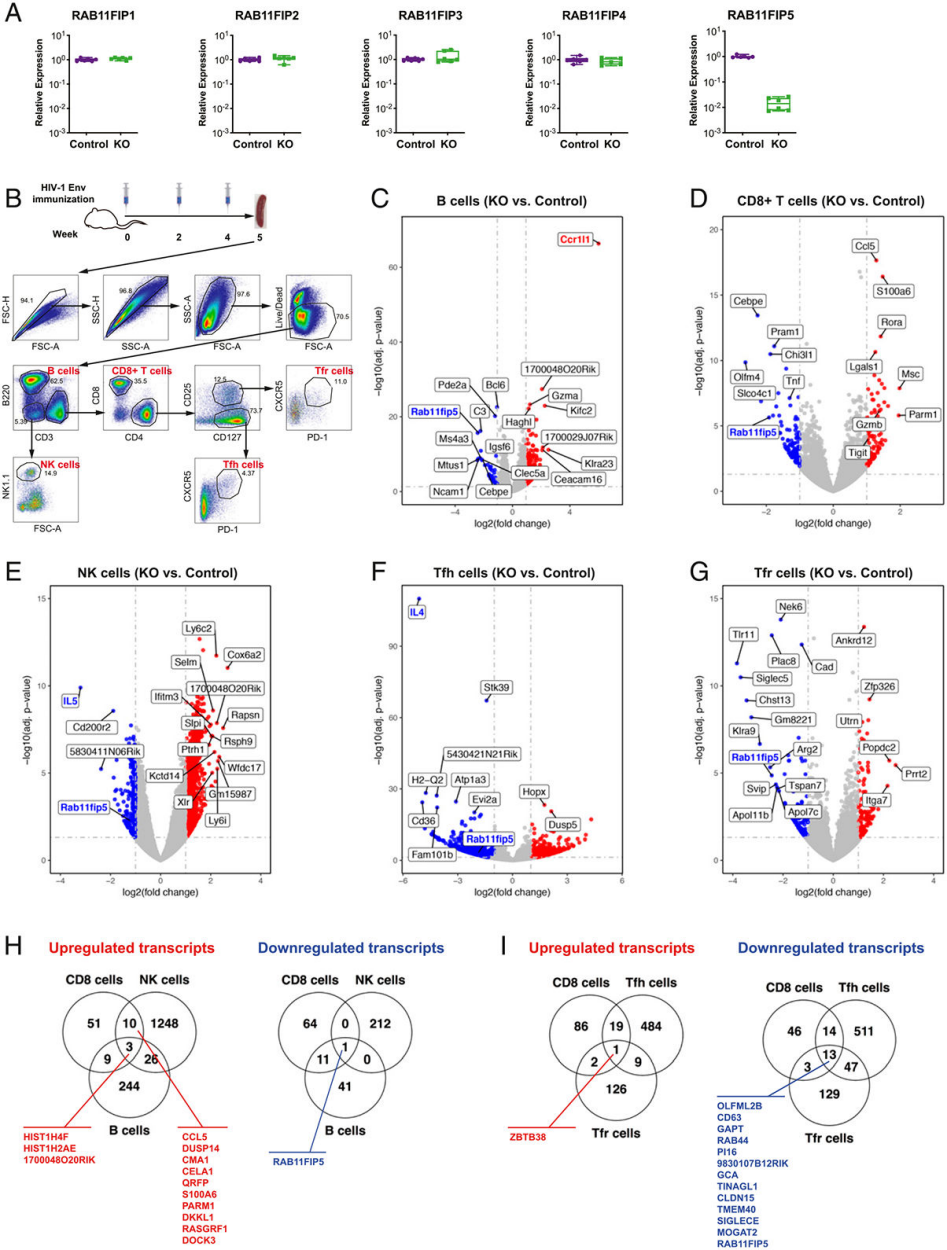
Transcriptome analysis of immune cell subsets in immunized *RAB11FIP5*^−/−^ mice. (**A**) Validation of *RAB11FIP5* KO in spleen samples from *RAB11FIP5*^−/−^ mice (KO; *n* = 6) and *RAB11FIP5*^fl/fl^ mice (Control; *n* = 6) by qPCR. Expression of mRNAs encoding *RAB11FIP5* and all the other *RAB11FIPs* in six animals per group is shown. Each dot represents data from one animal. Expression was calculated relative to that of the *ACTB* gene and normalized to the mean expression of the control group. Samples below the detection limit of the qPCR assay were assigned a cycle threshold value of 40. (**B**) Vaccination regimen and sorting strategies. *RAB11FIP5*^−/−^ (KO) mice (*n* = 4) or *RAB11FIP5*^fl/fl^ control mice (*n* = 4) were immunized with HIV-1 Env vaccine + GLA-SE adjuvant three times. Splenocytes were isolated and stained, and different cell types were sorted for RNA-Seq analysis of gene expression signatures. Gating strategy for FACS sorting B cells, CD8^+^ T cells and NK cells, CD4^+^ follicular helper T (Tfh) cells, and CD4^+^ follicular regulatory T (Tfr) cells is shown. (**C–G**) Gene expression profiles of B cells (C), CD8^+^ T cells (D), NK cells (E), CD4^+^ Tfh cells (F), and CD4^+^ Tfr cells (G) are shown as volcano plots. Genes with adjusted *p* < 0.05 and log2 (fold change) >1 were considered significant and are labeled in blue (downregulation) or red (upregulation). (**H**) Venn diagram showing common genes changed in B, CD8^+^ T, and NK cells. (**I**) Venn diagram showing genes changed in T cells (CD8^+^ T, CD4^+^ Tfh, and CD4^+^ Tfr cells).

We obtained mice with constitutive *RAB11FIP5* deletion of exon 2 by breeding *RAB11FIP5*^fl/fl^ mice with CMV-Cre transgenic mice. Although in the *RAB11FIP5*^fl/fl^ mice, exon 2 of the *RAB11FIP5* gene is flanked by loxP sites, homozygous *RAB11FIP5*^fl/fl^ remain viable, fertile, and produce normal levels of Rab11Fip5 protein (9). Therefore, *RAB11FIP5*^fl/fl^ mice were used as controls in this study. By crossing *RAB11FIP5*^fl/fl^ mice with CMV-Cre transgenic mice (Supplemental Fig. 1), constitutive *RAB11FIP5*^−/−^ mice were generated with the floxed exon 2 being excised, which abrogated the production of functional *RAB11FIP5* mRNA. The expression of *RAB11FIP5* mRNA was undetectable in spleen cells from the *RAB11FIP5*^−/−^ mice (Fig. 1A), confirming the deficiency of Rab11Fip5 in this mouse strain.

To investigate whether the deficiency of Rab11Fip5 resulted in alterations in the frequency of immune cell subsets, we analyzed the basal immune phenotype in *RAB11FIP5*^−/−^ and *RAB11FIP5*^fl/fl^ control mice. Compared with controls, *RAB11FIP5*^−/−^ mice had similar spleen and body weights (Supplemental Fig. 2A, 2B) as well as identical B, T, and NK cell subsets in their spleen, lymph nodes, and bone marrow (Supplemental Fig. 2C-F). Thus, these data show that the Rab11Fip5 KO does not alter the numbers of major immune cell subsets in this mouse model.

To identify Rab11Fip5-associated transcriptional changes in the context of a vaccine-induced immune response, we immunized *RAB11FIP5*^−/−^ mice and *RAB11FIP5*^fl/fl^ control mice with a HIV-1 Env vaccine and performed RNA-Seq on B cells, NK cells, CD8^+^ T cells, CD4^+^ Tfh cells, and CD4^+^ Tfr cells sorted from the immunized mice (Fig. 1B). Several significantly (*p* < 0.05) changed genes that were more than 2-fold increased or decreased were identified in *RAB11FIP5*^−/−^ mice (Fig. 1C-G). The most significantly upregulated gene in *RAB11FIP5*^−/−^ mice was *CCR1L1* in B cells (Fig. 1C), whereas the most significantly downregulated genes were *IL4* and *IL5* in Tfh and NK cells, respectively (Fig. 1E, 1F). *RAB11FIP5* was downregulated in all cell subsets, consistent with KO of *RAB11FIP5* (Fig. 1H, 1I). Ten genes were significantly upregulated in both NK cells and CD8^+^ T cells, including the transcripts that encode the chemokine *CCL5*, dual-specificity phosphatases *DUSP14*, and chymotryptic serine proteinases *CMA1* and *CELA1* (Fig. 1H). The only transcript upregulated in all three types of T cells (Tfh cells, Tfr cells, and CD8^+^ T cells) examined was a transcription factor *ZBTB38*, whereas 13 genes were significantly downregulated, including genes encoding CD63 and Rab44 that have vesicle trafficking functions (19, 20), as well as *SIGLEC-E*, which is the murine homologue for a human inhibitory immune checkpoint SIGLEC-9 (21) (Fig. 1I). Moreover, there was no significant change in the expression of Rab11 family genes (*RAB11A* and *RAB11B*) or RAB11FIP genes other than *RAB11FIP5* (*RAB11FIP1/2/3/4*) (Supplemental Fig. 3). Thus, a series of cytokine-related transcriptomic signatures were found in lymphocyte cell subsets from Rab11Fip5^−/−^ mice after vaccination, suggesting that Rab11Fip5 plays a role in cytokine production in immune responses.

### Characterization of Rab11Fip5-associated transcriptional signatures in B cells and NK cells

**FIGURE 2.**
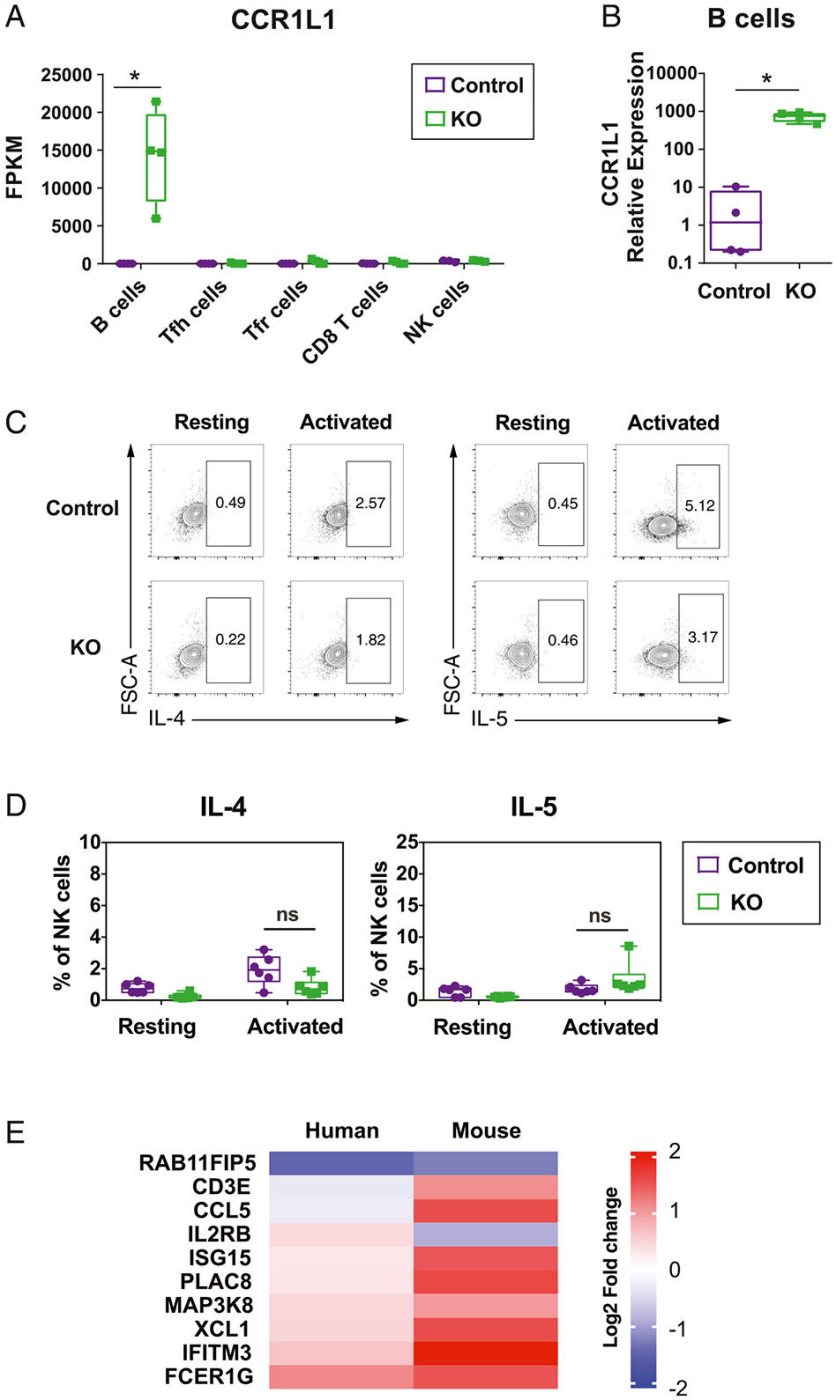
Characterization of Rab11Fip5-associated transcriptional signatures in B cell and NK cells. (**A** and **B**) CCR1L1 expression in immunized *RAB11FIP5*^−/−^ (KO) mice or *RAB11FIP5*^fl/fl^ control mice shown as fragments per kb of transcript per million mapped reads in different cell types (A) and relative expression as determined by qPCR in B cells (B). (**C** and **D**) Effect of *RAB11FIP5* deficiency on IL-4 and IL-5 production in mouse NK cells following stimulation with PMA + ionomycin. Mouse spleen cells were stimulated with 500 ng/ml PMA and 5 mg/ml ionomycin for 4 h in the presence of the protein transport inhibitor monensin. Intracellular staining was performed after stimulation. Representative example of the IL-4 and IL-5 intracellular staining (C) and percentages of IL-4 and IL-5 positive subsets (D) were shown. Each dot indicates one animal. The statistical significance of differences between groups in (B) and (C) was determined by Mann–Whitney *U* test. *p < 0.05; ns, not significant. (**E**) Comparison of Rab11Fip5-associated transcriptional changes in human NK cells and mouse NK cells. In humans, we studied NK cells from HIV-infected individuals expressing different levels of *RAB11FIP5* in PBMC, and transcriptional expression fold change indicates comparison between NK cells from subjects expressing low levels of *RAB11FIP5* (*RAB11FIP5*^low^) versus high levels of *RAB11FIP5* (*RAB11FIP5*^high^) in NK cells (*n* = 4/group) (11), whereas for mice, we studied NK cells isolated from HIV-1 Env-immunized animals and compared *RAB11FIP5* KO cells with control cells (*n* = 6/group). Significantly (*p* < 0.05) upregulated or downregulated genes are shown as a heat map.

We next validated the RNA-Seq transcriptome data by qPCR. In B cells, we confirmed that the transcript encoding the chemokine receptor CCR1L1 was upregulated in *RAB11FIP5*^−/−^ mice (Fig. 2A, 2B). However, although we observed downregulated *IL5* transcript expression in NK cells from HIV-1 Env-immunized *RAB11FIP5*^−/−^ mice by RNA-Seq (Fig. 1E), intracellular staining revealed that when splenocytes from *RAB11FIP5*^−/−^ mice were stimulated ex vivo with PMA/ionomycin there, the proportion of NK cells producing detectable levels of IL-5 protein were not significantly different to that in control mice (Fig. 2C, 2D). These data suggest that the reduced levels of *IL5* transcript expression in NK cells from immunized mice may be secondary to alterations in the function of other cell types in *RAB11FIP5*^−/−^ mice.

In human primary NK cells, we previously identified a series of differentially expressed transcripts in NK cells that expressed *RAB11FIP5* compared with NK cells with undetectable *RAB11FIP5* by single-cell RNA-Seq (11). To examine Rab11Fip5-associated transcriptional signatures that might overlap between human and mouse NK cells, we compared transcripts that were significantly altered (*p* < 0.05) with *RAB11FIP5* expression in both human and mouse NK cells. When *RAB11FIP5* expression is downregulated, six out of nine transcripts were upregulated in both human *RAB11FIP5^low^* and mouse *RAB11FIP5*^−/−^ NK cells, including genes encoding ISG15, PLAC8, MAP3K8, XCL1, IFITM3, and FCER1G (Fig. 2E). These data indicated that the Rab11Fip5-associated transcriptional signatures obtained from mouse *RAB11FIP5*^−/−^ NK cells are similar to data found in human NK cells with low *RAB11FIP5* expression. Notably, both the human and mouse NK cells studied were derived from an environment where *RAB11FIP5* was differentially expressed not only in NK cells but also other immune system cell subsets, so these data may reflect effects of Rab11Fip5 in NK cells themselves and/or secondary to effects of Rab11Fip5 on the function of other cell types that are common to both species.

### Analysis of the functional capacity of NK cells from RAB11FIP5^−/−^ mice

**FIGURE 3.**
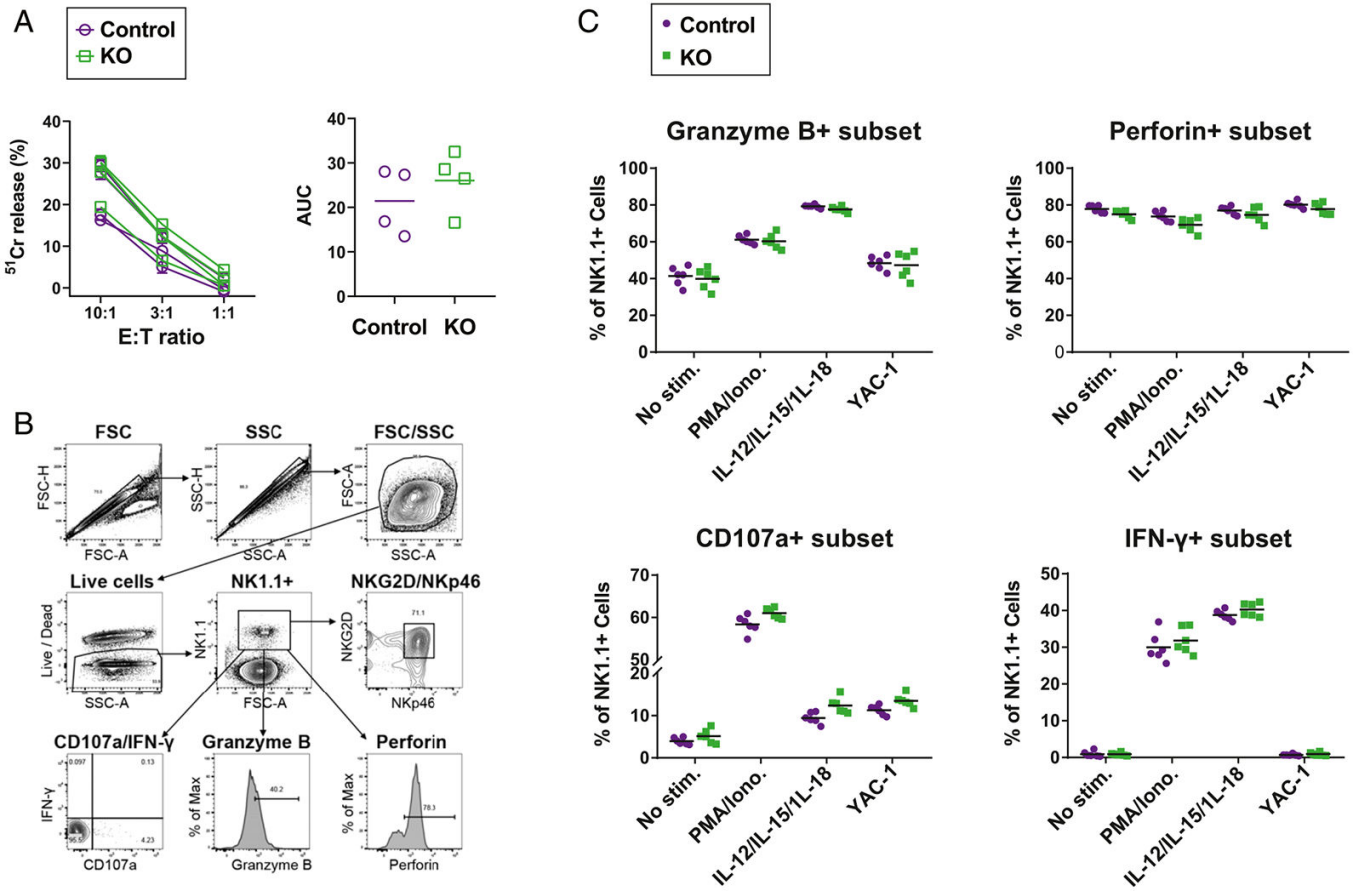
Characterization of NK cell function in *RAB11FIP5*^−/−^ mice. (**A**) NK cell cytotoxicity as measured by ^51^Cr release assay. NK cells were isolated by negative selection from splenocytes of *RAB11FIP5*^−/−^ mice (*n* = 4) and control *RAB11FIP5*^fl/fl^ mice (*n* = 4), stimulated with IL-15 for 12 h, and incubated with ^51^Cr-labeled YAC-1 target cells for 4 h at the indicated E:T ratios. Specific lysis of YAC-1 cells was analyzed by the measurement of supernatant ^51^Cr counts. Comparison of NK cell cytotoxicity of *RAB11FIP5*^−/−^ mice and control *RAB11FIP5*^fl/fl^ mice (data points from each animal are shown as connected dots [left], and area under the curve [AUC] values are also plotted [right]). AUC were calculated using GraphPad. Bar represents the mean value of each group. Each symbol represents data from a given animal, and representative data from two independent experiments are shown. (**B** and **C**) Perforin/granzyme and cytokine levels in mouse NK cells. Spleen cells of *RAB11FIP5*^−/−^ mice (*n* = 6) and control *RAB11FIP5*^fl/fl^ mice (*n* = 6) were stimulated with PMA + ionomycin, cytokines (IL-12, IL-15, and IL-18), or YAC-1 target cells for 4 h in the presence of CD107a Ab and the protein transport inhibitor monensin. Surface staining was performed with a NK cell marker NK1.1. Subsequently, intracellular staining was performed with IFN-γ, granzyme B, and perforin Abs. CD107a, granzyme B, perforin, and IFN-γ expression were analyzed by gating on NK1.1^+^ cells (B) and shown as percentages of positive subsets (C). Each dot represents data from one animal.

Our previous study showed that NK cells from bnAb individuals have upregulated *RAB11FIP5* expression and altered NK cell functions (11), and we also confirmed a direct effect of Rab11Fip5 expression on NK cell function by overexpressing *RAB11FIP5* in a human NK cell line. To examine whether Rab11Fip5 expression is required for NK cell function, we examined cytolysis and cytokine production by NK cells from *RAB11FIP5*^−/−^ mice. Primary NK cells were isolated from *RAB11FIP5*^−/−^ mice and *RAB11FIP5*^fl/fl^ mice, respectively, and analyzed for cytotoxicity against YAC-1 lymphoma cells. No significant differences were observed in the killing activity (Fig. 3A). We next measured degranulation and cytokine production in splenic NK cells upon stimulation. The degranulation marker CD107a, cytokine IFN-β, perforin, and granzyme B production were similar in NK cells from *RAB11FIP5*^−/−^ and *RAB11FIP5*^fl/fl^ mice (Fig. 3B, 3C). Thus, Rab11Fip5 is not required for the function of NK cells from *RAB11FIP5*^−/−^ mice.

### Correlation of RAB11FIP5 expression with TNF-α secretion in CD8^+^ T cells

**FIGURE 4.**
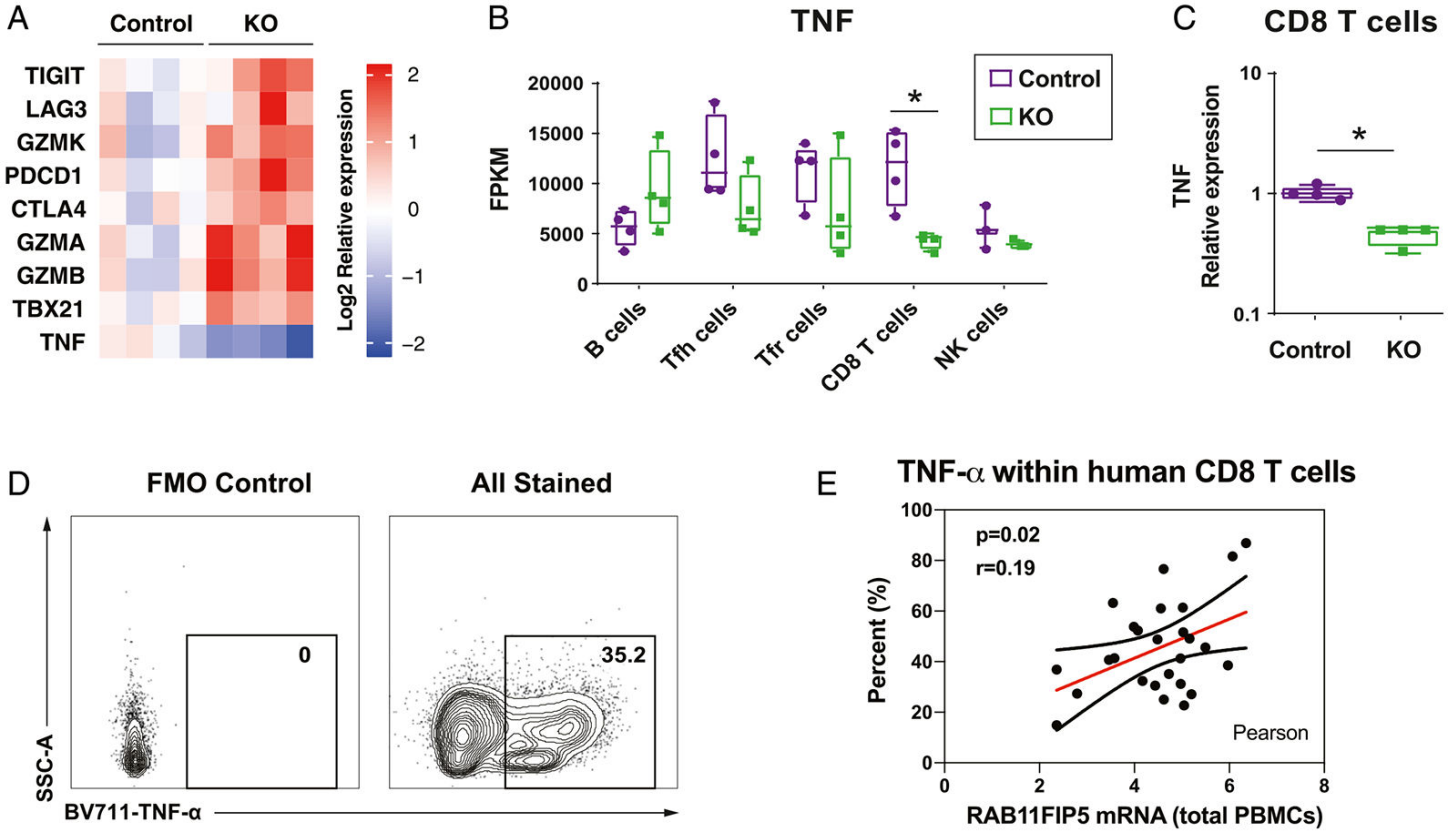
Rab11Fip5 expression is associated with TNF-α production in CD8^+^ T cells. (**A**) Comparison of expression of genes encoding key immune checkpoints, cytokines, and cytotoxic proteases in CD8^+^ T cells from immunized *RAB11FIP5*^−/−^ mice and immunized control mice, shown as a heat map. *TIGIT, LAG3, CTLA4*, and *PDCD1* are the genes encoding immune checkpoint markers TIGIT, LAG-3, CTLA-4, and PD-1, respectively. *TBX21* is the gene encoding T-Box Transcription Factor 21 (TBX21), also known as T-bet. *GZMA, GZMB*, and *GZMK* represent genes encoding granzyme A, B, and K. *TNF* is the gene encoding the cytokine TNF-α. (**B** and **C**) TNF expression in different cell types from immunized *RAB11FIP5*^−/−^ mice or control *RAB11FIP5*^fl/fl^ mice shown as fragments per kb of transcript per million mapped reads (FPKM) (B) and relative expression as determined by qPCR in CD8^+^ T cells (C). (**D** and **E**) TNF-α production by CD8^+^ T cells correlates with *RAB11FIP5* mRNA expression in human PBMC. PBMC from 26 subjects were activated with anti-CD3/CD28 Abs for 3 d and then stimulated with PMA + ionomycin for 5 h before intracellular staining for TNF-α. (D) Representative example of intracellular staining for TNF-α (gated on CD3^+^CD4^−^ T cells); the TNF-α^+^ subset was defined based on a fluorescence minus one control. (E) Pearson analysis of the correlation between the percentage of TNF-α^+^ cells within CD8 T cells and *RAB11FIP5* mRNA expression in PBMC (*n* = 26). Each dot represents data from an individual donor. Lines depict fit line and 95% confidence bands. The statistical significance of differences between groups was determined by Mann–Whitney *U* test. *p < 0.05.

Genes expressing multiple well-characterized immune checkpoint markers (TIGIT, LAG-3, PD-1, and CTLA4), T-bet, and cytotoxic effector molecules (granzymes A, B, and K) were more highly expressed in CD8^+^ T cells from immunized *RAB11FIP5*^−/−^ than control mice (Fig. 4A), suggesting that *RAB11FIP5*^−/−^ CD8^+^ T cells may have been more highly activated than those from control animals. However, TNF-α transcripts were significantly lower in CD8^+^ T cells from HIV Env-immunized mice lacking Rab11Fip5 (Fig. 4B, 4C). We also evaluated TNF-α expression in CD8^+^ T cells following in vitro anti-CD3/CD28 stimulation of PBMCs from 26 HIV-infected human subjects in whom we had previously measured PBMC *RAB11FIP5* expression levels (Fig. 4D, 4E). Importantly, we found a significant positive correlation between *RAB11FIP5* mRNA levels in PBMCs and TNF-α production in activated CD8^+^ T cells (*p* = 0.02, Pearson correlation). These data in mice and humans suggest that Rab11Fip5 may regulate TNF-α production in CD8^+^ T cells.

### Rab11Fip5 expression is associated with IL-4 production in CD4^+^ T cells

**FIGURE 5.**
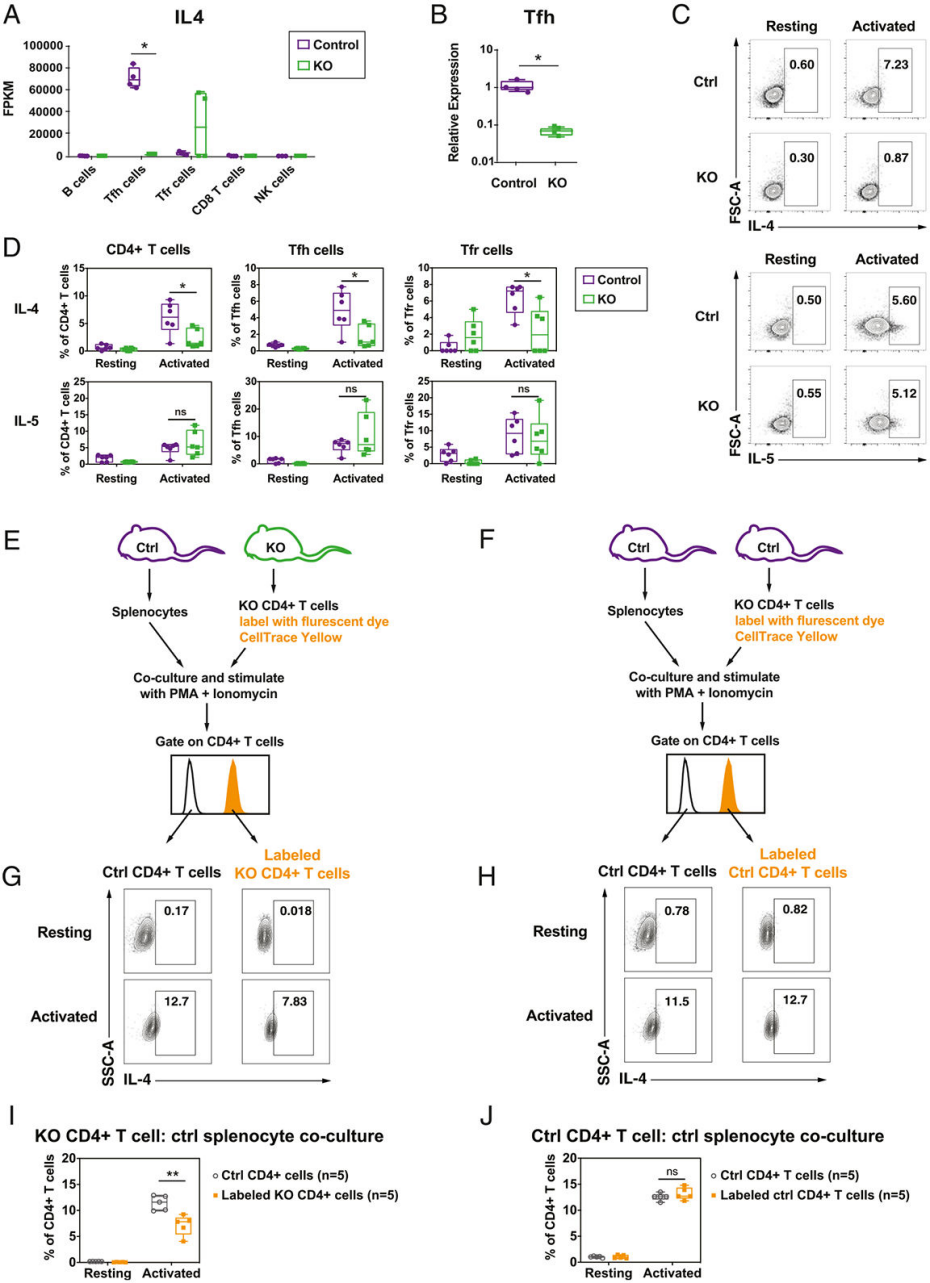
Rab11Fip5 expression is associated with IL-4 production in CD4^+^ T cells. (**A** and **B**) IL-4 expression in different cell types from immunized *RAB11FIP5*^−/−^ (KO) mice or control *RAB11FIP5*^fl/fl^ (Control) mice shown as fragments per kb of transcript per million mapped reads (FPKM) (A) and relative expression in Tfh cells, as determined by qPCR analysis (*p < 0.05, as determined by Mann–Whitney *U* test) (B). (**C** and **D**) Effect of Rab11Fip5 deficiency on IL-4 and IL-5 production by mouse CD4^+^ T cells following stimulation. Mouse spleen cells were stimulated with PMA and ionomycin in the presence of the protein transport inhibitor monensin. Intracellular staining was performed after stimulation. Representative example of the IL-4 and IL-5 intracellular staining (C) and percentages of IL-4 and IL-5 positive subsets (D) were shown. Each dot indicates one animal, and the statistical significance of differences between groups was determined by Mann–Whitney *U* test. *p < 0.05. (**E** and **F**) Coculture of *RAB11FIP5*^−/−^ (KO) CD4^+^ cells (E) or *RAB11FIP5*^fl/fl^ control CD4^+^ cells (F) with littermate control splenocytes. CD4^+^ T cells were isolated from spleen cells of KO mice or control mice using magnetic beads, labeled with CellTrace Yellow dye, and mixed with control mice spleen cells. The KO CD4^+^ T cell–control splenocyte mixture (E), as well as the control CD4^+^ T cell–control splenocyte mixture (F), were cocultured, stimulated for either 4 h or overnight (two independent experiments), and analyzed for IL-4 production by intracellular staining. The results were similar in the two independent experiments. (**G**–**J**) IL-4 production in the stimulated KO CD4^+^ T cell–control splenocyte coculture or the stimulated control CD4^+^ T cell–control splenocyte coculture. Two independent experiments with five animals in each group were performed. Representative examples of the IL-4 intracellular staining (G and H) and percentages of IL-4 positive subsets (I and J) were compared between CellTrace Yellow–labeled and unlabeled CD4^+^ T cells. Each dot indicates one animal, and the statistical significance of differences between groups was determined by Mann–Whitney *U* test. **0.001 < *p* < 0.01. ns, not significant.

In our previous study, higher *RAB11FIP5* expression was also observed in CD4^+^ T cells from individuals developing bnAbs compared with those who did not make bnAbs (11). In mice, we found that *IL4*, which encodes the Th2 cytokine IL-4, was expressed at significantly lower levels in CD4^+^ Tfh cells from HIV Env-immunized *RAB11FIP5*^−/−^ as compared with similarly immunized control animals (Fig. 5A, 5B). By intracellular staining, we confirmed that IL-4 protein production following PMA + ionomycin stimulation was lower in CD4^+^ Tfh cells as well as total CD4+ T cells from *RAB11FIP5*^−/−^ mice (Fig. 5C, 5D). To determine if the trait of low IL-4 production by *RAB11FIP5*^−/−^ CD4^+^ T cells is dependent on other *RAB11FIP5*^−/−^ cells or rather is the trait intrinsic to *RAB11FIP5*^−/−^ CD4 T cells, we isolated splenic CD4^+^ T cells from *RAB11FIP5*^−/−^ mice or control *RAB11FIP5*^fl/fl^ mice, labeled with CellTrace Yellow fluorescent dye, and cocultured them with control *RAB11FIP5*^fl/fl^ spleen cells (Fig. 5E, 5F). After stimulation of the cocultured cells with PMA+ionomycin, in the *RAB11FIP5*^−/−^ CD4^+^ T cell–control splenocyte group, *RAB11FIP5*^−/−^ CD4^+^ cells showed reduced IL-4 production phenotype compared with the cocultured control CD4^+^ cells (Fig. 5G, 5H), whereas no difference was observed in the control–control CD4^+^ T cell coculture group (Fig. 5I, 5J). These data demonstrated that at both transcriptome and protein levels, Rab11Fip5 deficiency was associated with reduced IL-4 expression in mouse CD4^+^ T cells, and this IL-4–related phenotype is an intrinsic property of the *RAB11FIP5*^−/−^ CD4^+^ cells and not dependent on other splenic cells in *RAB11FIP5*^−/−^ mice.

### HIV-1–neutralizing Ab responses in RAB11FIP5^−/−^ mice following Env vaccination

**FIGURE 6.**
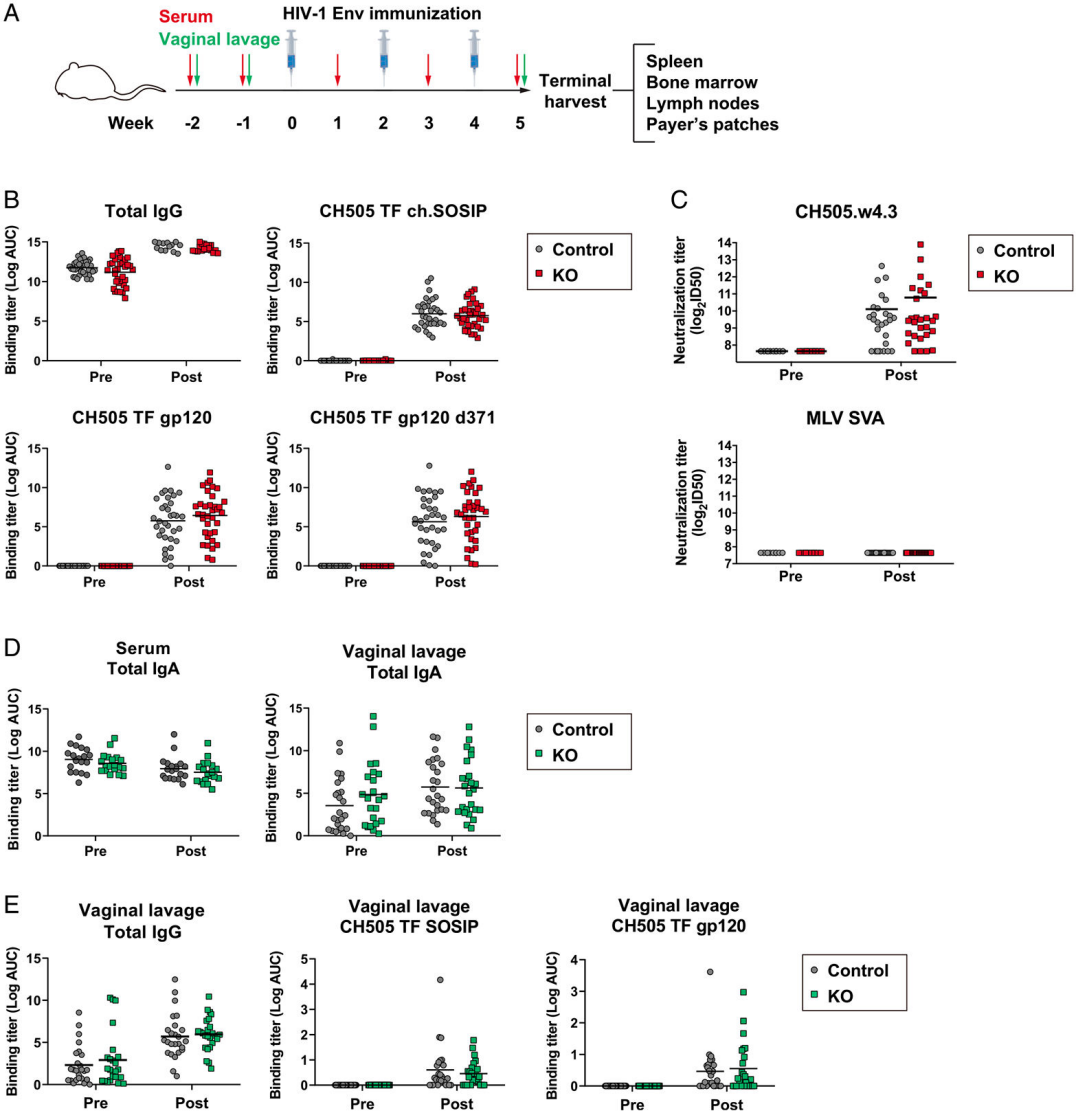
Systemic and mucosal Ab responses elicited by vaccination in *RAB11FIP5*^−/−^ mice. (**A**) Vaccination regimen. *RAB11FIP5*^−^ mice and control *RAB11FIP5*^fl/fl^ mice were immunized three times with unstabilized CH505 TF chimeric (ch) SOSIP. Serum and vaginal lavage samples were collected at the indicated time points before and after immunization. (**B** and **C**) Rab11Fip5 is not required for HIV-1 Env vaccine-induced Ab responses. (B) Serum IgG HIV Env immunogen ELISA binding titers in *RAB11FIP5*^−/−^ mice (*n* = 36) and control RAB11FIP5^fl/fl^ mice (*n* = 34). Pre- and postimmunization IgG titers against CH505 TF gp120, CH505 TF gp120 d371, CH505 TF ch.SOSIP, and total IgG titers are shown. (C) Serum neutralization activity against CH505.w4.3 pseudovirus and a control salmonid alphavirus–murine leukemia virus pseudovirus. Combined data from four independent immunization studies are shown. The statistical significance of differences between the two groups was determined by Mann–Whitney *U* test, and no significant differences were found. (**D** and **E**) Rab11Fip5 is not required for IgA transcytosis or IgG movement to mucosal sites in vivo. Vaginal lavage samples were collected pre- and postimmunization from female *RAB11FIP5*^−/−^ mice (*n* = 25) and control *RAB11FIP5*^fl/fl^ mice (n = 25). (D) Quantification of serum IgA and secretory IgA by ELISA at pre- and postvaccination time points. (E) ELISA binding titers for total and HIV Env-specific IgG. Each dot indicates data from one animal. Combined data from three independent immunization studies are shown. The statistical significance of differences between the two groups was determined by Mann–Whitney *U* test, and no significant differences were found.

*RAB11FIP5* has been shown to be a key signature that is related to the presence of HIV-1 bnAbs in individuals chronically infected with HIV (11). To gain insight into the role of Rab11Fip5 in Ab responses, we compared the humoral response elicited following HIV-1 Env vaccination in *RAB11FIP5*^−/−^ and control mice. Animals were immunized with HIV-1 chimeric SOSIP trimer proteins derived from the CH505 TF Env formulated in the TLR 4 agonist adjuvant GLA-SE (16). After three injections (Fig. 6A), Abs to Env subunit gp120 and SOSIP trimer proteins in serum were elicited to similar levels in these two mouse strains, with no differences observed in total serum IgG levels (Fig. 6B). *RAB11FIP5*^−/−^ mice exhibited only a nonsignificant trend for higher average tier 1 (neutralization-sensitive) virus neutralization titers (Fig. 6C). To examine vaccine-induced B cell responses, we also compared the number of HIV-1 specific GL7^+^CD95^+^ germinal center (GC) B cells, CD38^low^IgG^+^ GC B cells, and IgG^+^ memory cells. We found no differences in bone marrow, spleen, lymph node, or Peyer’spatch of these B cell subsets (Supplemental Fig. 4A, 4B). Thus, the lack of Rab11Fip5 in mice did not alter the magnitude or quality of Ab responses following HIV-1 Env vaccination.

### Rab11Fip5 is not required for IgA transcytosis or IgG movement to mucosal sites in vivo

A previous study found that knockdown of Rab11Fip5 led to decreased transcytosis of polymeric IgA (pIgA) in MDCK cells (5). We examined total IgA Ab titers in both serum and vaginal lavage before and after vaccination and found that *RAB11FIP5*^−/−^ mice had comparable serum and secretory IgA levels to those of control mice (Fig. 6D). Similarly, no difference was observed in mucosal total IgG as well as in mucosal gp120- or SOSIP-specific IgG titers between *RAB11FIP5*^−/−^ mice and control mice (Fig. 6E). These data indicate that deficiency of Rab11Fip5 does not abrogate IgA transcytosis or IgG movement to mucosal sites.

### Rab11Fip5 deficiency does not alter susceptibility to influenza virus challenge

**FIGURE 7.**
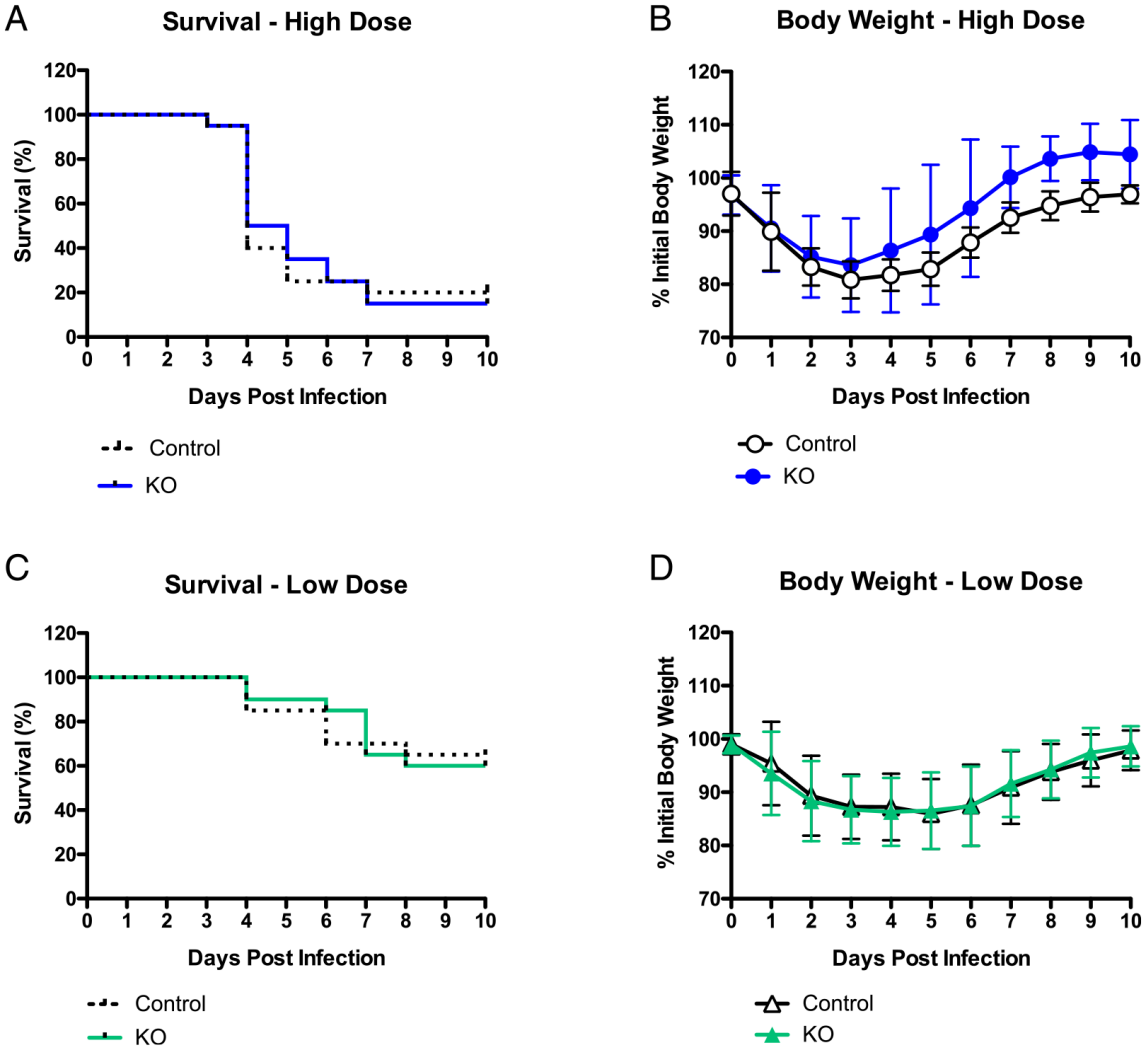
Influenza H3N2 virus X-31 challenge. *RAB11FIP5*^−^ mice (*n* = 20) and *RAB11FIP5*^fl/fl^ control mice (*n* = 20) were intranasally challenged with a high dose (10^6^ TCID50) or low dose (10^4^ TCID50) influenza X-31 virus. Survival and body weight were monitored daily for 10 d. Animals that reached the body weight cutoff (80% of original weight) were euthanized. Combined data from two independent immunization studies are shown. (**A**) Survival curves of high dose influenza X-31 virus–infected mice. (**B**) Body weight of high-dose influenza X-31 virus–infected mice. (**C**) Survival curves of low-dose influenza X-31 virus–infected mice. (**D**) Body weight of low-dose influenza X-31 virus–infected mice.

Given our previous observation that the overexpression of *RAB11FIP5* increased proinflammatory cytokine levels in a human NK cell line (11), we determined whether Rab11Fip5 deficiency in mice could change the susceptibility to influenza virus infection, which is associated with the degree of cytokine storm (22, 23). *RAB11FIP5*^−/−^ mice and *RAB11FIP5*^fl/fl^ controls were infected with influenza virus strain X-31 (H3N2) using a high or low dose. No significant differences were found in survival rates or body weights between groups (Fig. 7A-D), suggesting that the absence of Rab11Fip5 did not alter resistance to influenza virus infection in vivo.

## DISCUSSION

In this study, we have studied the role of the Rab11-recycling endosome effector Rab11Fip5 in a KO mouse model. We previously found that transcripts encoding *RAB11FIP5* are significantly higher in PBMCs from HIV-infected individuals who develop bnAbs, with elevated *RAB11FIP5* expression being observed in cell subsets including NK cells, CD8^+^ T cells, and CD4^+^ T cells (11). In this study, we identified key transcriptional signatures in lymphocyte subsets from HIV-1 Env-immunized Rab11Fip5-deficient mice and found that Rab11Fip5 may regulate the production of cytokines, including TNF-α in CD8^+^ T cells and IL-4 in CD4^+^ T cells. Other observations made in Rab11Fip5 mice indicated that Rab11Fip5 is not required for NK cell functions, elicitation of HIV-1 Abs, or survival from influenza infection, although it may have roles in these processes that can be substituted for by other class I Fips. However, these findings support an essential role for Rab11Fip5-associated recycling endosomes in certain cytokine secretion pathways.

It is known that Rab11-positive recycling endosomes are essential for the formation of immunological synapses, the contact regions formed between lymphocytes and cells with which they mediate cognate interaction (24). Immunologic synapses are formed between CD8^+^ T cells or NK cells and target cells with infected or transformed or between CD4^+^ T cells and B cells when help is provided (25). Notably, a previous study showed that Rab11 colocalizes with IFN-γ– and TNF-α–containing but not with perforin-containing granules in NK cells (14), suggesting that recycling endosomes may also modulate cytokine secretion but not NK cell cytotoxic granule release. However, the Rab11FIP(s) involved in the delivery of Lck to the plasma membrane for signaling following NK cell and TCR triggering (24) and transport of cytokines to the cytoplasm for release (14) were not characterized.

Our results revealed that CD8^+^ T cells from immunized *RAB11FIP5*^−/−^ mice had markedly reduced *TNF* transcripts, and our observation that PBMC *RAB11FIP5* mRNA levels in HIV-infected subjects were correlated with TCR ligation-stimulated TNF-α production in CD8^+^ T cells suggested a role for Rab11fip5 in CD8^+^ T cell TNF-α responses in humans as well as in mice. The reduction in TNF-α production by CD8^+^ T cells in the context of Rab11Fip5 deficiency could reflect an essential role of Rab11Fip5 within T cells and/or may be secondary to effects of Rab11Fip5 in other cell types. However, our previous observation that Rab11Fip5 overexpression in a human NK cell line enhanced production of IFN-γ (11) indicate that endogenous Rab11Fip5 is involved in the regulation of lymphocyte production of at least certain cytokines. Interestingly, we found that NK cells from *RAB11FIP5*^−/−^ mice did not exhibit defects in cytotoxic activity or IFN-γ production, which may indicate that other Fips are able to substitute for at least some aspects of Rab11Fip5 activity in murine NK cells. It is known that Rab11Fip5 shares highly homologous N-terminal C2 phospholipid-binding domain and C-terminal Rab11-binding domain with Rab11Fip1 and Rab11Fip2 (26), which allow class I Rab11Fips (Rab11Fip1/2/5) interacting with the same group of proteins and localizing in the same subcellular compartments (26, 27). Although we and others (9) speculated that other class I Rab11 FIPs could compensate the lack of Rab11Fip5, the expression of *RAB11FIP1* and *RAB11FIP2* were not altered as measured by qPCR (Supplemental Fig. 2). This phenomenon has also been described by Bacaj and colleague in their study on neuronal functions of Rab11Fip5 (9). It is possible that class I FIPs of Rab11 are redundant at protein level, and the levels of Rab11Fip1 or Rab11Fip2 proteins, without being upregulated, may be able to compensate the function of Rab11Fip5. Nevertheless, further studies using KO models with Rab11Fip1, Rab11Fip2, and Rab11Fip5 deletion alone or in combination will be needed to test this hypothesis.

The mechanism(s) by which Rab11Fip5 influences cytokine production in lymphocytes are unclear, but it seems likely that roles for Rab11Fip5 in intracellular trafficking of multiple different proteins may be involved. Prior studies have identified roles for Rab11 recycling endosomes in the transport of cytokines (and also newly synthesized perforin) from the Golgi to the cell surface (24) and in delivery of VAMPs to the plasma membrane where they facilitate vesicle fusion to enable content release (28, 29), so Rab11Fip5 may be involved in cytokine secretion. However, our observation of reduced levels of TNF and IL-4 transcripts within activated CD8^+^ and CD4^+^ T cells, respectively, indicates the effects on cytokine induction, which could be multifactorial, given the involvement of recycling endosomes in a plethora of cellular functions (30, 31). For example, the involvement of Rab11 recycling endosomes in the delivery of Lck to the plasma membrane (24) may impact on TCR triggering. Given that CD8^+^ T cell functions are triggered in a hierarchical fashion, with TNF-α upregulation requiring strong signaling (32), attenuation of TCR signaling in the context of Rab11Fip5 deficiency could have a particularly pronounced effect on *TNF* expression.

Results from experiments performed with human primary CD8^+^ T cells suggested that the *RAB11FIP5*^−/−^ mouse model may mirror aspects of cytokine regulation in T cells. Moreover, in NK cells, when we compared the Rab11fip5-associated transcriptomic changes in mouse (*RAB11FIP5*^−/−^ NK cells versus *RAB11FIP5*^fl/fl^ NK cells) and human (RAB11FIP5^low^ NK cells versus *RAB11FIP5*^high^ NK cells), we found that six out of nine significantly changed transcripts were common to both human *RAB11FIP5*^low^ and mouse *RAB11FIP5*^−/−^ NK cells. Whether these alterations were due to NK cell–intrinsic effects of Rab11Fip5 deficiency and/or were secondary to effects of low Rab11Fip5 on the function of other cell types requires further investigation. Additionally, we found that *CCR1L1* mRNA was upregulated in mouse *RAB11FIP5*^−/−^ B cells after vaccination; further study is needed to understand the biological function of *CCR1L1*, if it is involved in humoral immune responses, and whether there is an analogous Rab11Fip5-regulated gene in human B cells.

The finding that *RAB11FIP5* is expressed at higher levels in PBMCs from HIV-infected subjects who develop bnAbs (11) suggests that Rab11Fip5 may play a role in the induction of protective humoral responses. However, Rab11Fip5 deletion in mice did not alter their susceptibility to influenza virus challenge, and these mice mounted normal Ab responses to vaccination with HIV-1 Env immunogens that elicit conventional Ab responses lacking neutralization breadth. These observations suggest that in our previous human study, elevated *RAB11FIP5* levels may either be required for creating the unique immunological environment that is required to support the development of Abs with the unusual characteristics of HIV-1 bnAbs (33) or that they may not be a primary determinant for induction of HIV-1–neutralizing Abs but rather may be a marker for other key immunological traits, such as NK cell dysfunction, which we hypothesize may contribute to bnAb induction by reducing NK cell–mediated constraints on CD4^+^ Tfh availability to support GC responses (11).

The phosphorylation of Rab11Fip5 has been reported to be required for transcytosis of pIgA in cell lines (5). Therefore, we initially expected that the pIgA secreted into the mucosal sites should be lower in *RAB11FIP5*^−/−^ mice. In contrast, we did not observe differences in mucosal IgA or IgG levels in the *RAB11FIP5*^−/−^ mice. Hence, it is possible that when Rab11Fip5 is absent, other class I Fip molecules (4) are able to mediate pIgA movement into mucosal sites.

Thus, data presented in this study demonstrate the potential regulatory roles of Rab11Fip5 in cytokine immune responses. Future work will focus on dissecting the cellular and molecular mechanisms by which Rab11Fip5-associated recycling endosomes contribute to the formation of immunological synapses and regulating cytokine production within lymphocyte subsets, especially those entailed in the regulation of CD8^+^ T cell TNF-α and CD4^+^ T cell IL-4 production.

## Supplementary Material

SUPPLEMENTAL FIGURES

## Abbreviations used in this article:

AF: Alexa Fluor
AUC: area under curve
BD: Becton Dickinson
bnAb: broadly neutralizing Ab
BV: Brilliant Violet
^51^Cr: chromium-51
Env: envelope
GC: germinal center
KO: knockout
pIgA: polymeric IgA
qPCR: quantitative PCR
Rab11Fip: Rab11 family–interacting protein
RNA-Seq: RNA sequencing
TCID50: median tissue culture infective dose
TF: transmitted founder
Tfh: T follicular helper
Tfr: T follicular regulatory
