# Supplementary material for: RAB11FIP5-Deficient Mice Exhibit Cytokine-Related Transcriptomic Signatures
Source: Immunohorizons. Author manuscript; Available in PMC 2021 Apr 16. (PMC8050958; doi:10.4049/immunohorizons.2000088)
Supplement: SUPPLEMENTAL FIGURES [file NIHMS1688260-supplement-SUPPLEMENTAL_FIGURES.pdf]

SUPPLEMENTAL FIGURES

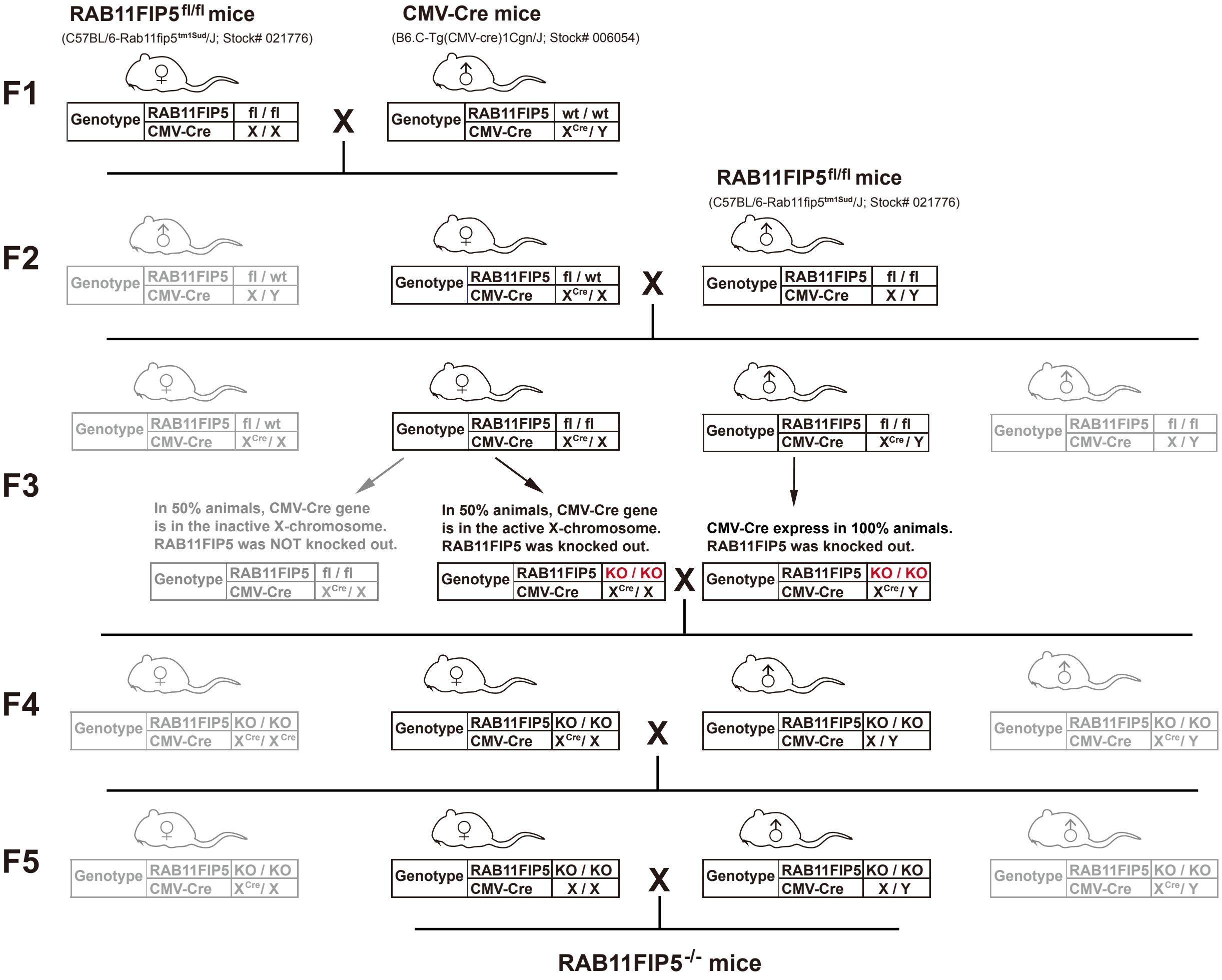

Figure S1. Generation of constitutive *RAB11FIP5* knockout (*RAB11FIP5*$^{-/-}$) mice. *RAB11FIP5*$^{-/-}$ mice were generated by breeding *RAB11FIP5* floxed mice (*RAB11FIP5*$^{fl/fl}$) with CMV-Cre mice. In the *RAB11FIP5*$^{fl/fl}$ conditional mutant mouse, exon 2 of the *RAB11FIP5* gene is flanked by loxP sites. Mice homozygous for this allele are viable, fertile and produce normal levels of Rab11Fip5 protein. When crossed with a Cre-expressing strain, the floxed exon 2 is excised and therefore the expression of functional *RAB11FIP5* mRNA is abolished. In our study, as the CMV-Cre transgene is X-chromosome linked, F1 female *RAB11FIP5*$^{fl/fl}$ mice were crossed with male CMV-Cre mice. F2 female littermates were kept and bred with *RAB11FIP5*$^{fl/fl}$ mice. In F3 approximately half of the female littermates still had the *RAB11FIP5* gene due to X-chromosome inactivation in the CMV-Cre positive allele, while males and the remaining females had *RAB11FIP5* deletion. By inbreeding F3 *RAB11FIP5*$^{-/-}$ male and females, we eventually generated constitutive *RAB11FIP5*$^{-/-}$ mice independent of Cre. All experiments in this study were conducted with mice after F5.

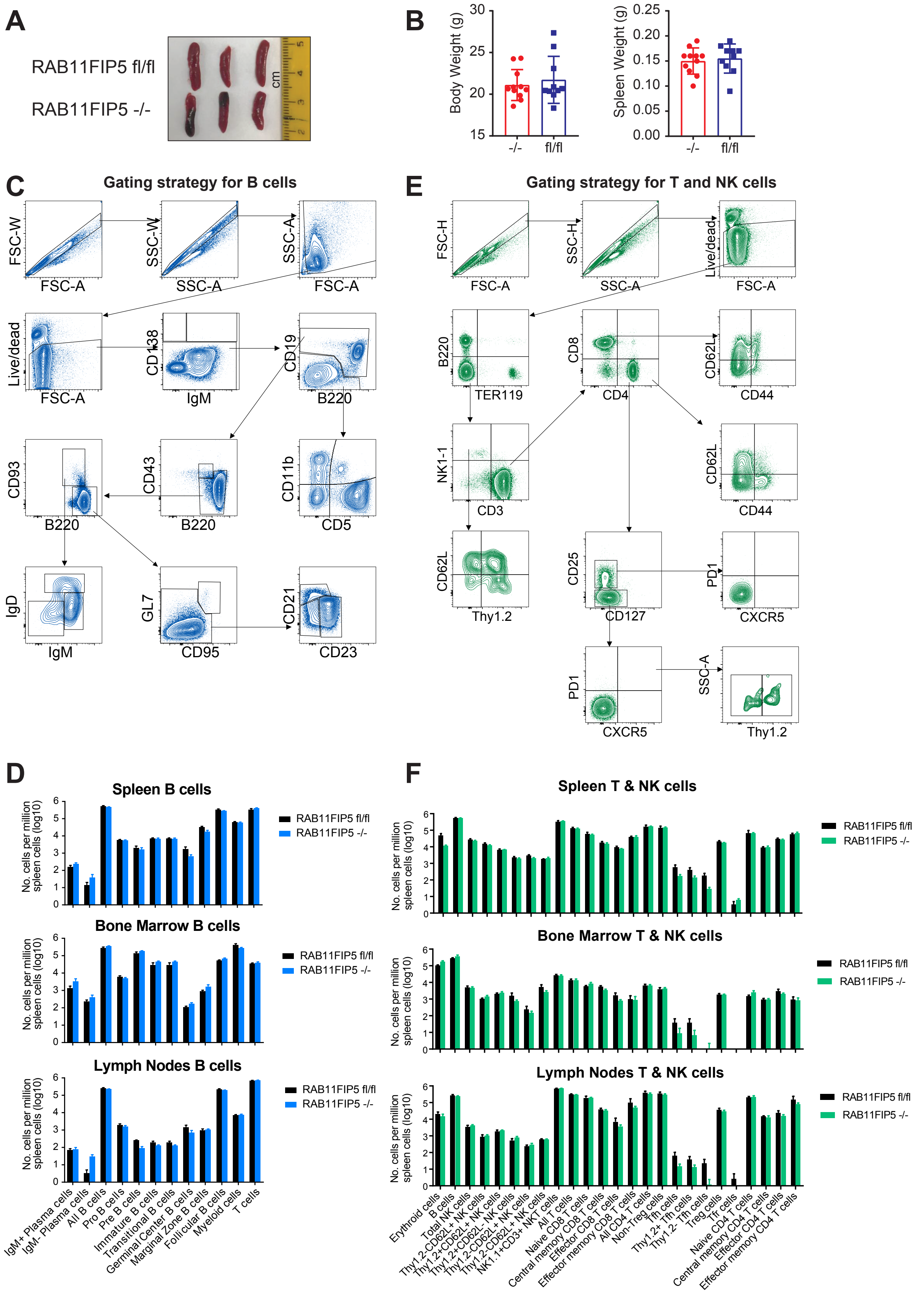
A
RAB11FIP5 fl/fl
RAB11FIP5 -/-
cm
B
Body Weight (g)
Spleen Weight (g)
-/-
fl/fl
C
Gating strategy for B cells
FSC-W
FSC-A
SSC-W
SSC-A
Live/dead
CD138
IgM
CD19
B220
CD93
CD43
CD11b
CD5
IgD
GL7
CD95
CD21
CD23
E
Gating strategy for T and NK cells
FSC-H
SSC-H
TER119
CD8
CD4
CD62L
CD44
NK1-1
CD3
Thy1.2
CD25
CD127
PD1
CXCR5
D
Spleen B cells
Bone Marrow B cells
Lymph Nodes B cells
No. cells per million spleen cells (log10)
RAB11FIP5 fl/fl
RAB11FIP5 -/-
IgM+ Plasma cells
IgM- Plasma cells
All B cells
Pro B cells
Pre B cells
Immature B cells
Transitional B cells
Germinal Center B cells
Marginal Zone B cells
Follicular B cells
Myeloid cells
T cells
F
Spleen T & NK cells
Bone Marrow T & NK cells
Lymph Nodes T & NK cells
Erythroid cells
B cells
Total NK cells
Thy1.2-CD62L+ NK cells
Thy1.2+CD62L+ NK cells
Thy1.2+CD62L- NK cells
Thy1.2-CD62L- NK cells
NK1.1+CD3+ NKT cells
All T cells
Naive CD8 T cells
Central memory CD8 T cells
Effector CD8 T cells
Effector memory CD8 T cells
All CD4 T cells
Non-Treg cells
Tfh cells
Thy1.2+ Tfh cells
Thy1.2- Tfh cells
Treg cells
Tfr cells
Naive CD4 T cells
Central memory CD4 T cells
Effector CD4 T cells
Effector memory CD4 T cells

Figure S2. No major perturbations in lymphocyte subsets in *RAB11FIP5*$^{-/-}$ mice.
(A) Pictures of spleens of representative *RAB11FIP5*$^{-/-}$ and control *RAB11FIP5*$^{fl/fl}$ mice.
(B) Body and spleen weights of *RAB11FIP5*$^{-/-}$ (*n*=11) and control *RAB11FIP5*$^{fl/fl}$ mice (*n*=10).
(C-D) Phenotypic analysis of B cells in *RAB11FIP5*$^{-/-}$ (*n*=3) and control *RAB11FIP5*$^{fl/fl}$ (*n*=3) mice. (C) Gating strategy for B cell subsets. (D) Cells from lymph nodes, spleen, and bone marrow were analyzed and the bar graphs show the absolute number of total cells, IgM^{+} plasma cells (PC; CD138^{+}), IgM^{-} PC, total B cells (CD19^{+}B220^{+}), pro B cells (CD^{+}B220^{+}), pre-B cells (IgM^{-}IgD^{-}), immature (Imm) B cells (B220^{+}CD93^{+}IgM^{+}IgD^{-}), transitional (Trans) B cells (B220^{+}CD93^{+}IgM^{+}IgD^{+}/low), germinal center (GC) B cells (B220^{+}CD93^{-}GL7^{+}CD95^{+}CD38^{low}), marginal zone (MZ) B cells (B220^{+}CD93^{-}GL7^{-}CD95^{-}IgG^{-}CD21^{high}CD23$^{low/-}$), follicular (Fo) B cells (B220^{+}CD93^{-}GL7^{-}CD95^{-}IgG^{-}CD21^{int}CD23^{high}), myeloid cells (IgM^{-}CD19^{-}B220^{-}CD11b^{+}CD5^{-}), and T cells (IgM^{-}CD19^{-}B220^{-}CD11b^{-}CD5^{+}).
(E-F) Phenotypic analysis of T cells and NK cells in *RAB11FIP5* $^{-/-}$ and fl/fl mice. (E) Gating strategy for T cell and NK cell subsets. (F) Cells from lymph node, spleen and bone marrow were analyzed and the bar graphs show the absolute number of total cells, erythroid cells (TER119^{+}B220^{-}), B cells (TER119^{-}B220^{+}), total NK cells (CD3^{-}NK1.1^{+}), Thy1.2^{-}CD62L^{+} NK cells, Thy1.2^{+}CD62L^{+} NK cells, Thy1.2^{+}CD62L^{-} NK cells, Thy1.2^{-}CD62L^{-} NK cells, total T cells (CD3^{+}), CD8^{+} T cells, naive CD8^{+} T cells (CD8^{+}CD44^{-}CD62L^{+}), central memory (CM) CD8^{+} T cells (CD8^{+}CD44^{+}CD62L^{+}), effector CD8^{+} T cells (CD8^{+}CD44^{+}CD62L^{-}), effector memory (EM) CD8^{+} T cells (CD8^{+}CD44^{-}CD62L^{-}), CD4^{+} T cells, non-Treg (CD4^{+}CD127^{high}CD25^{low}), Tfh (CD4^{+}CD127^{high}CD25^{low}CXCR5^{+}PD-1^{+}), Thy1.2^{-} Tfh, Thy1.2^{+} Tfh, Treg (CD4^{+}CD127^{low}CD25^{high}), Tfr (CD4^{+}CD127^{low}CD25^{high}CXCR5^{+}PD-1^{+}), naive CD4^{+} T cells (CD4^{+}CD44^{-}CD62L^{+}), central memory (CM) CD4^{+} T cells (CD4^{+}CD44^{+}CD62L^{+}), effector CD4^{+} T cells (CD4^{+}CD44^{+}CD62L^{-}), and effector memory (EM) CD4^{+} T cells (CD4^{+}CD44^{-}CD62L^{-}).

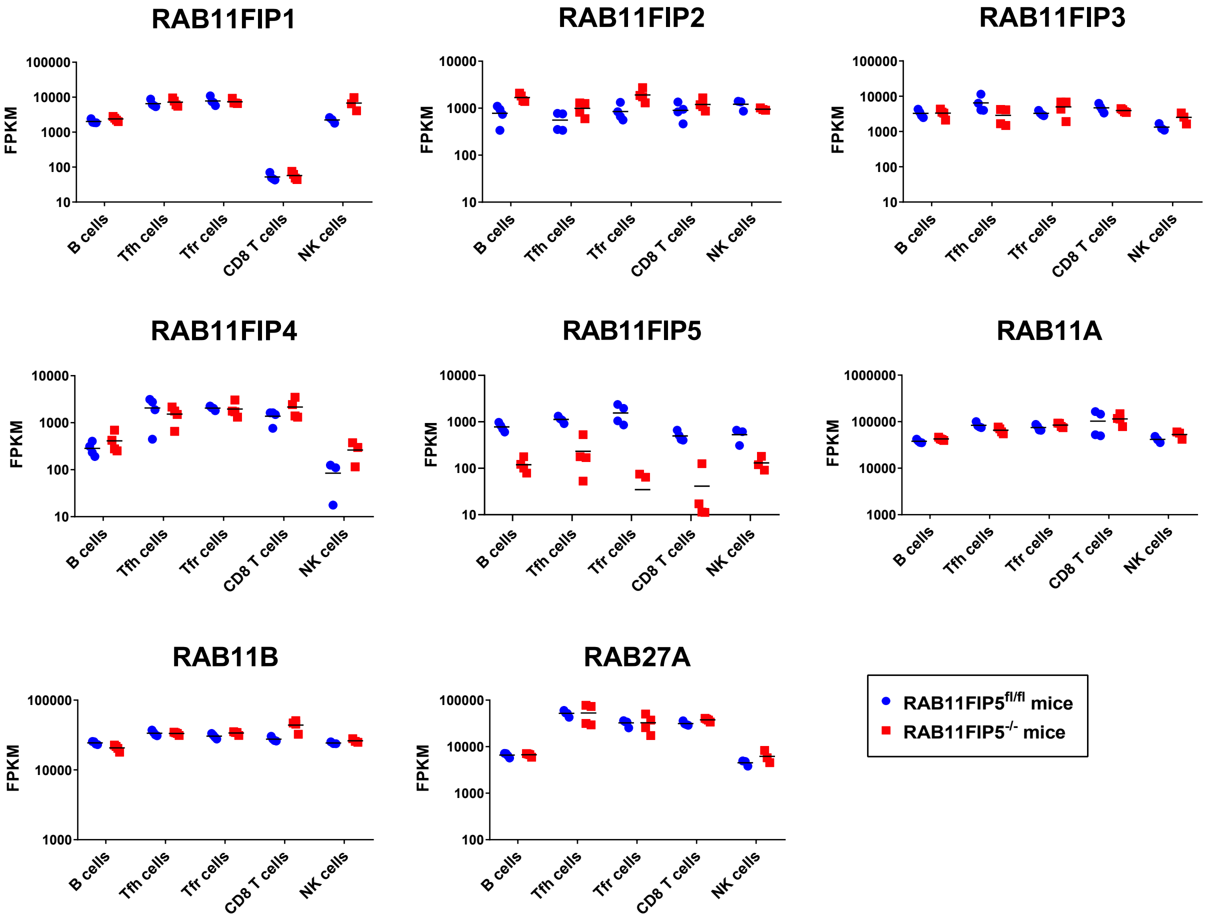

Figure S3. ***RAB11FIP5*** **deficiency has no effect on expression of other RAB11 family genes and RAB11 family interacting protein (Fip) genes.** FPKMs of *RAB11A*, *RAB11B*, and *RAB11FIPs* genes in B cells, Tfh cells, Tfr cells, CD8+ T cells and NK cells are shown as dots; each dot indicates data from one animal. While the deletion of exon 2 in *RAB11FIP5* alleles results in the absence of a complete and functional *RAB11FIP5* mRNA, incomplete/partial *RAB11FIP5* transcripts may still exist at various levels in different cell types as indicated by decreased FPKM in the $RAB11FIP5^{-/-}$ mice.

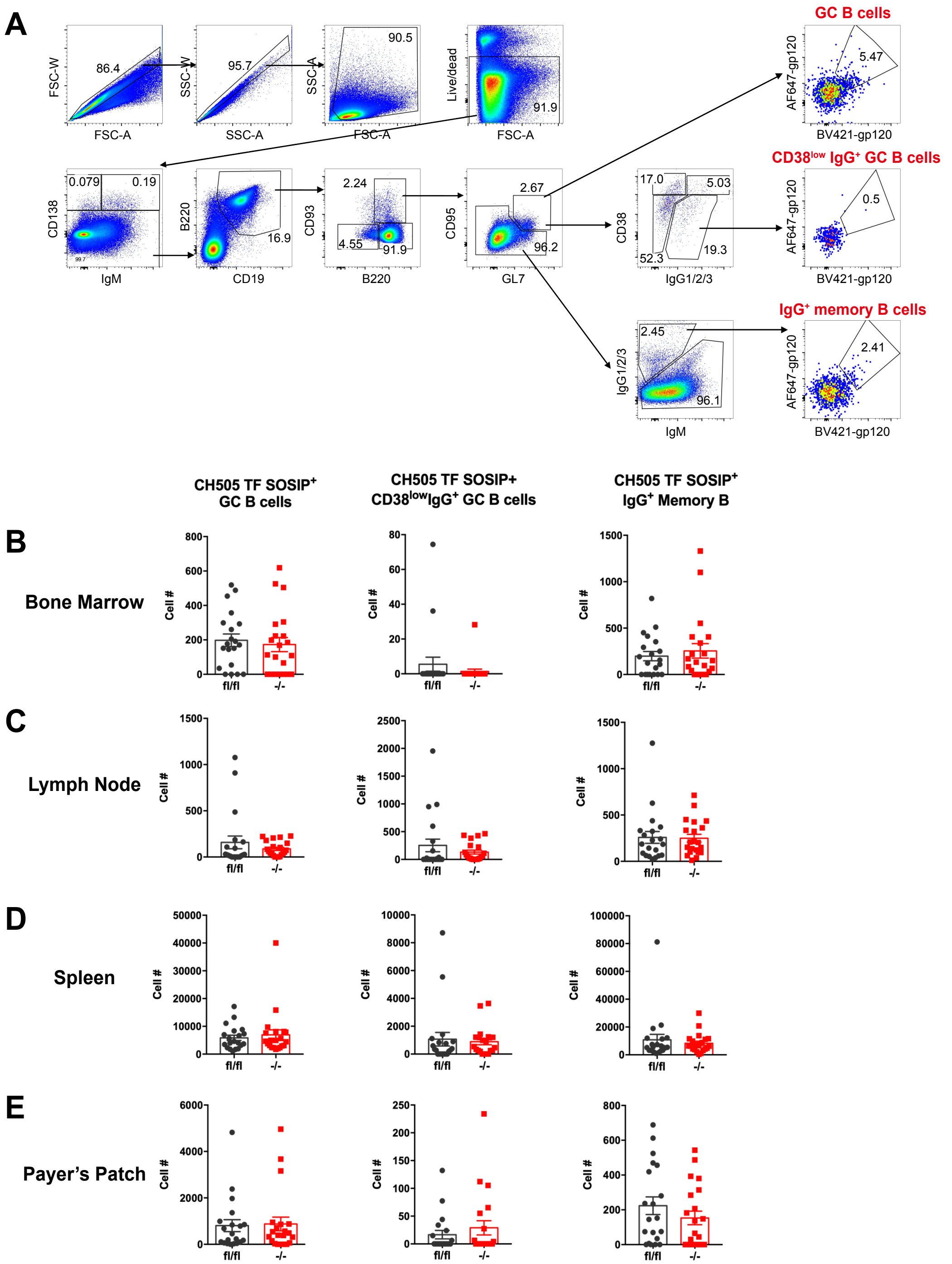

Figure S4. Analysis of antigen-specific B cell responses in immunized mice.

(A) Gating strategy to identify HIV-Env binding cells (double-positive for binding to CH505 gp120 tetramers labeled with two different fluorochromes) in germinal center (GC) B cells, CD38low IgG^{+} GC B cells, and IgG+ memory B cells.

(B-E) HIV-1 CH505 TF SOSIP-specific GC B cells, CD38^{low} IgG^{+} GC B cells, and memory B cells were compared between *RAB11FIP5*$^{-/-}$ mice (n=21) and control *RAB11FIP5*$^{fl/fl}$ mice (n=20) in different tissues. Cells from bone marrow (B), spleen (C), lymph node (D), and Peyer's patch (E) were analyzed and absolute numbers of SOSIP-binding cells are shown in each tissue. Bars represent mean ± SD and each dot indicates one animal. Combined data from three independent immunization studies are shown. The statistical significance of differences between groups was determined by Mann-Whitney U test, and no significant differences were found.
